# 3D atlas of tinamou (Neornithes: Tinamidae) pectoral morphology: Implications for reconstructing the ancestral neornithine flight apparatus

**DOI:** 10.1111/joa.13919

**Published:** 2023-06-26

**Authors:** Klara E. Widrig, Bhart‐Anjan S. Bhullar, Daniel J. Field

**Affiliations:** ^1^ Department of Earth Sciences University of Cambridge Cambridge UK; ^2^ Department of Earth and Planetary Sciences Yale University New Haven Connecticut USA; ^3^ Peabody Museum of Natural History Yale University New Haven Connecticut USA; ^4^ Museum of Zoology University of Cambridge Cambridge UK

**Keywords:** diceCT, flight, flightlessness, muscles, Palaeognathae, soft tissue reconstruction, Tinamidae

## Abstract

Palaeognathae, the extant avian clade comprising the flightless ratites and flight‐capable tinamous (Tinamidae), is the sister group to all other living birds, and recent phylogenetic studies illustrate that tinamous are phylogenetically nested within a paraphyletic assemblage of ratites. As the only extant palaeognaths that have retained the ability to fly, tinamous may provide key information on the nature of the flight apparatus of ancestral crown palaeognaths—and, in turn, crown birds—as well as insight into convergent modifications to the wing apparatus among extant ratite lineages. To reveal new information about the musculoskeletal anatomy of tinamous and facilitate development of computational biomechanical models of tinamou wing function, we generated a three‐dimensional musculoskeletal model of the flight apparatus of the extant Andean tinamou (*Nothoprocta pentlandii*) using diffusible iodine‐based contrast‐enhanced computed tomography (diceCT). Origins and insertions of the pectoral flight musculature of *N. pentlandii* are generally consistent with those of other extant volant birds specialized for burst flight, and the entire suite of presumed ancestral neornithine flight muscles are present in *N. pentlandii* with the exception of the biceps slip. The pectoralis and supracoracoideus muscles are robust, similar to the condition in other extant burst‐flying birds such as many extant Galliformes. Contrary to the condition in most extant Neognathae (the sister clade to Palaeognathae), the insertion of the pronator superficialis has a greater distal extent than the pronator profundus, although most other anatomical observations are broadly consistent with the conditions observed in extant neognaths. This work will help form a basis for future comparative studies of the avian musculoskeletal system, with implications for reconstructing the flight apparatus of ancestral crown birds and clarifying musculoskeletal modifications underlying the convergent origins of ratite flightlessness.

## Graphical abstract

Tinamous are the only members of Palaeognathae (a clade containing giant flightless birds such as the ostrich) that are capable of flight. We utilized contrast enhanced micro CT scanning to explore in detail the musculoskeletal anatomy of the flight apparatus of the Andean tinamou, *Nothoprocta pentlandii*.

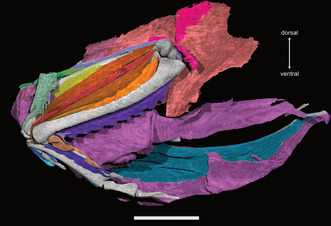

## 1 INTRODUCTION

The avian crown group (Neornithes) includes over 10,000 living species (Billerman et al., 2020). This clade is divided into the reciprocally monophyletic groups Palaeognathae and Neognathae, with Neognathae (=Galloanserae + Neoaves) comprising over 99% of extant bird diversity (Phillips et al., 2009). By contrast, the diversity of extant palaeognaths is comparatively low. Today, Palaeognathae is represented by 46 species of tinamous (Tinamidae), two species of ostriches (Struthionidae), two species of rheas (Rheidae), the monotypic emu and three species of cassowaries (Casuariidae), and approximately five species of kiwi (Apterygidae; Billerman et al., 2020; Winkler et al., 2020b). Crown Palaeognathae also encompasses substantial extinct diversity; for example, nine species of moa (Dinornithiformes) and up to four species of elephant birds (Aepyornithidae), which went extinct during the Holocene (Bunce et al., 2009; Grealy et al., 2023; Hansford et al., 2018). Total‐group Palaeognathae also includes the extinct and apparently volant Lithornithidae from the Palaeocene and Eocene of Europe and North America (Houde, 1986, 1988; Houde & Olson, 1981; Nesbitt & Clarke, 2016; Parris & Hope, 2002; Stidham et al., 2014; Widrig & Field, 2022; Yonezawa et al., 2017). Palaeognaths are diagnosed by numerous anatomical features including a distinctive palatal structure with enlarged basipterygoid processes, as well as rhamphothecal grooves, and open ilioischiadic foramina (Bock, 1962; Cracraft, 1974; Parkes & Clark, 1966; Pycraft, 1900). Some distinctive features of palaeognaths that have long been considered crown bird plesiomorphies, such as fusion of the pterygoid‐palatine complex (Pycraft, 1900) instead appear to represent hitherto overlooked palaeognath synapomorphies in light of recent fossil discoveries (Benito, Kuo, et al., 2022; Torres et al., 2021).

Tinamous are notable among extant palaeognaths as the only group that has retained the ability to fly, albeit only over relatively short distances (Winkler et al., 2020a). Tinamous are predominantly ground‐dwelling, and will fly in short bursts to escape predators or to roost in low‐hanging branches (Forshaw, 1991; Winkler et al., 2020a). These birds have an exclusively neotropical distribution and occupy a wide range of habitats in Central and South America, from dense forest to open grassland (Winkler et al., 2020a). Fossils recognizable as crown group tinamous appear abruptly in the early Miocene (16.5 mya), in the Pinturas and Santa Cruz formations of southern Patagonia (Bertelli & Chiappe, 2005; Chandler, 2012; Chiappe, 1991). The monophyly of Tinamidae is well established, and their internal phylogenetic relationships are generally well understood (Almeida et al., 2021; Bertelli, 2017; Bertelli et al., 2014; Porzecanski, 2004).

Until relatively recently, the phylogenetic position of Tinamidae with respect to the remainder of Palaeognathae was less certain. Tinamidae was traditionally thought to represent the sister taxon of the flightless palaeognaths, which were united in a monophyletic ratite clade (Ratitae) by features such as the lack of a triosseal canal and sternal keel, and possession of a fused scapulocoracoid (Cracraft, 1974; McGowan, 1982). This interpretation was upheld by most phylogenetic analyses of morphological data (Bourdon et al., 2009; Lee et al., 1997; Livezey & Zusi, 2007), though those that focused on cranial characters recovered alternative relationships (Elzanowski, 1995; Johnston, 2011). Because ratites are all found on landmasses in the Southern Hemisphere (ostriches in Africa, rheas in South America, kiwi and moa in New Zealand, emu in Australia, cassowaries in Australia and New Guinea, and elephant birds in Madagascar), prevailing wisdom held that ratites shared a flightless common ancestor and diverged through vicariance driven by the breakup of the supercontinent Gondwana (Cracraft, 1973; 2001).

However, over the last 20 years, a host of molecular phylogenetic studies have illustrated that this straightforward interpretation of palaeognath evolution is untenable. Molecular phylogenetic analyses examining both nuclear (Almeida et al., 2021; Baker et al., 2014; Chojnowski et al., 2008; Grealy et al., 2017; Hackett et al., 2008; Harshman et al., 2008; Smith et al., 2012; Wang et al., 2021) and mitochondrial (Grealy et al., 2017; Mitchell et al., 2014; Phillips et al., 2009; Urantówka et al., 2020) loci recover a phylogenetic position for tinamous nested deeply within the ratites, rendering Ratitae paraphyletic and suggesting that large size and flightlessness have been acquired several times independently throughout palaeognath evolutionary history. This hypothesis has since been corroborated by numerous large‐scale phylogenomic analyses (e.g., Cloutier et al., 2019; Feng et al., 2020; Jarvis et al., 2014; Kimball et al., 2019; Prum et al., 2015; Sackton et al., 2019; Takezaki, 2023; Yonezawa et al., 2017). In studies incorporating ancient DNA derived from recently extinct taxa, tinamous have been recovered as the sister taxon of New Zealand's moa (Almeida et al., 2021; Baker et al., 2014; Cloutier et al., 2019; Grealy et al., 2017; Mitchell et al., 2014; Phillips et al., 2009; Smith et al., 2012; Takezaki, 2023; Urantówka et al., 2020; Yonezawa et al., 2017), contrary to earlier hypotheses that placed moa and kiwi as sister taxa and upheld the notion that these groups diverged after a flightless ancestor became isolated on New Zealand (Cracraft, 1974). Meanwhile, ancient DNA studies also support a sister‐group relationship between kiwi and the elephant birds of Madagascar, a landmass that has been isolated since the early Cretaceous (Ali & Krause, 2011; Grealy et al., 2017; Mitchell et al., 2014). Phylogenomic divergence time analyses based on concatenated sequences of nuclear and mitochondrial genes suggests that the moa–tinamou divergence took place long after the geographic isolation of New Zealand. Yonezawa et al. (2017) estimated this divergence to have taken place between 56.1 and 50.2 mya, Grealy et al. (2017) placed it between 59.4 and 45.5 mya, and Almeida et al. (2021) found divergence dates between 58.9 and 43.7 mya depending on the constraints used, though it is possible that this divergence was even more recent (Berv & Field, 2018).

These recent molecular phylogenetic topologies imply that the major extant clades of flightless ratites are all descended from flying ancestors capable of dispersing considerable distances, with flightlessness arising among paleognaths independently at least six times, and gigantism arising independently a minimum of five times (Grealy et al., 2017; Mitchell et al., 2014). However, it is difficult to definitively reject the hypothesis that the ancestors of tinamous re‐acquired flight from a flightless ancestor. Indeed, this interpretation provides a more parsimonious solution than the hypothesis of numerous independent flight losses among ratites, albeit one that seems improbable in light of several lines of evidence including the propensity for repeated independent flight losses in other bird clades (e.g., Rallidae: Gaspar et al., 2020; Livezey, 2003). Moreover, a comparison of rates of wing development in tinamou, ostrich, and emu embryos suggests that different heterochronic mechanisms were responsible for transitions to flightlessness in ostriches and emu, further bolstering the hypothesis that ratites have undergone multiple independent losses of flight capacity (Bickley & Logan, 2014; Farlie et al., 2017; Faux & Field, 2017). The assumptions of Dollo's Law, which states that a complex trait such as flight is unlikely to be regained once lost (Collin & Miglietta, 2008; Simpson, 1953) favours the explanation of independent flight loss in ratite lineages (Phillips et al., 2009).

Given the prevailing hypothesis that the last common ancestor of extant palaeognaths was relatively small bodied and capable of flight, the biology of tinamous, as the only extant volant palaeognaths, emerges as a key source of information for understanding the ancestral biology of palaeognaths. All extant ratites have undergone major evolutionary alterations to their flight apparatus, including major reductions and rearrangements of ancestral flight muscles due to the loss of the sternal keel. The pectoralis muscle (hereafter M. pectoralis), which powers the downstroke of the wing during flight in volant birds, originates on the coracoid in most ratites as opposed to the sternum, as is the case in flying birds (Maxwell & Larsson, 2007). The wings of kiwi, cassowaries, and emus are so greatly reduced that they serve no major function, while moa lacked a forelimb skeleton entirely (Cracraft, 1974). Emus exhibit a loss of canalization in their appendicular musculature, as weak selective pressure on these vestigial structures has allowed for greater population‐level variation to arise (Maxwell & Larsson, 2007). A loss of functional constraints and the resultant absence of stabilizing selection may result in greater perturbations to ancestral morphologies over time, making ancestral states more difficult to reliably infer. Tinamous are therefore the only extant group in which a functional palaeognath flight apparatus can be studied in detail, and, given the sister‐group relationship between palaeognaths and all other extant birds, further insights into anatomy of the tinamou flight apparatus will enable stronger inferences regarding the flight apparatus of ancestral crown birds.

In this study, we imaged an Andean tinamou (*Nothoprocta pentlandii*) using diffusible iodine‐based contrast‐enhanced computed tomography (diceCT) and examined the resulting scans via digital dissection. This method has numerous advantages over traditional gross dissection (Gignac et al., 2016; Gignac & Kley, 2014; Lautenschlager et al., 2014), perhaps the most obvious of which is the fact that, unlike traditional dissection, it is non‐destructive, allowing for repeated analysis of the same dataset by any number of researchers while minimizing damage to the original specimen (Early et al., 2020). Traditional dissection methods are difficult to apply to small muscles (Baverstock et al., 2013; Cox & Jeffery, 2011), whereas digital dissection allows complex, delicate structures that would likely be damaged during gross dissection to be visualized in situ, preserving anatomical fidelity (Gignac & Kley, 2014). DiceCT allows for bones and muscles to be viewed three‐dimensionally, both individually and within their broader anatomical context. Most detailed descriptive works on avian anatomy have focused on domestic birds, and this promising new method has only been applied to a relatively small number of extant bird taxa to date (Bribiesca‐Contreras & Sellers, 2017; Lautenschlager et al., 2014; Li & Clarke, 2016; Sullivan et al., 2019).

Early studies of tinamou anatomy focused mainly on osteology and palatal morphology to investigate the relationship between ratites and tinamous (Lowe, 1928; 1942; McDowell, 1948; Parker, 1862, 1864, 1866; Pycraft, 1900). Those studies that included myology focused only on those muscles considered of taxonomic importance (Alix, 1874, 1876; Beddard, 1898; Fürbringer, 1902; Gadow & Selenka, 1891; Garrod, 1873, 1874, 1875; Lowe, 1928, 1942; Pycraft, 1900). At least two previous studies of the complete appendicular myology of tinamous were carried out using traditional gross dissection methods. Hudson et al. (1972) examined 22 specimens from 13 species in the genera *Crypturellus*, *Nothoprocta*, *Nothura*, *Eudromia*, and *Tinamotis*, and described myological data only. Suzuki et al. (2014) investigated two *Eudromia elegans* specimens for myology and four *E. elegans* and one *Nothoprocta cinerascens* as osteological specimens. Both of these studies examined the myology of the pelvic girdle in addition to the pectoral girdle. While the suite of muscles present in tinamids has been well established by these studies, tinamou musculature have never before been characterized in three dimensions, nor interpreted within a deeper evolutionary context involving inferences on the morphology of stem palaeognaths and the ancestral crown bird.

Here, we generated a detailed atlas of tinamou wing anatomy through three‐dimensional imaging of the musculoskeletal system of the flight apparatus of *Nothoprocta pentlandii*. We expect that this reconstruction will be useful for comparative anatomical studies across the avian crown group, and for use in future reconstructions of morphology in ancestral volant palaeognaths. The relevant morphology of Tinamidae has been suggested to be largely morphologically uniform across the group (Hudson et al., 1972); as such, we consider our investigation of the morphology of *N. pentlandii* as probably reflective of Tinamidae more broadly.

## 2 MATERIALS AND METHODS

### 2.1 Specimen

The *Nothoprocta pentlandii* specimen investigated here (YPM ORN 143617) was an adult female (sexed by DNA test) donated to the Yale Peabody Museum by the Sedgwick County Zoo in Wichita, Kansas, USA, after dying at the age of 2 years, 6 months of natural causes. It weighed 283.5 grams at death. The specimen was frozen prior to staining and injected with formalin as a fixative, before being stained in Lugol's solution made in 70% ethanol for approximately 5 weeks. The specimen was unpinioned (that is, its primary feathers were not clipped to prevent it from flying).

### 2.2 Data archiving

The data used in this study were archived on Morphosource (https://www.morphosource.org/projects/000529384).

### 2.3 Imaging

YPM ORN 143617 was scanned at the Harvard Biotomography Center on a Nikon XT H 225 ST scanner. Scanning parameters were: 90 kV, 85 μA, 1 second exposure, no frame averaging, 3141 views. The voxel size is 78 microns, and no unstained reference scan was taken.

### 2.4 3D model construction

The scans were digitally segmented, rendered, and imaged in VG Studio Max 3.3.0 (Volume Graphics GmbH). Each muscle and bone discussed herein was manually segmented using the “draw” tool. We manually segmented both bone and muscle from the same scan, as ethanol‐based staining solutions lead to less bone demineralization than water‐based solutions which facilitated the visualization of skeletal elements (Early et al., 2020). After segmentation was completed for each element, volumes were smoothed using the “smooth” tool at level 3.

### 2.5 Musculoskeletal nomenclature

Nomenclature follows Baumel and Witmer (1993), with English equivalents used for osteological terms when possible. In several instances, tendons of origin and insertion could not be fully visualized. In these cases, we refer to Hudson et al. (1972) and Suzuki et al. (2014), as a traditional dissection of the original specimen was outside the scope of this study. As most connective tissues were not visible in our contrast‐stained specimen, muscle origins and insertions on connective tissues could not be visualized.

## 3 RESULTS: ANATOMICAL DESCRIPTIONS

### 3.1 Osteology

#### 3.1.1 Scapula

At its cranial end, the scapula articulates with the coracoid at the coracoidal articular surface to form the glenoid cavity, which articulates with the humerus. The acromion projects cranially, forming the scapula's cranialmost tip. The scapula of *Nothoprocta* has a prominent pneumatic foramen, located on the ventromedial surface of the cranial end opposite to the humeral articular surface. The enlarged cranial end of the scapula tapers to meet the blade of the scapula at the scapular neck. The dorsoventral width of the scapular blade is largely consistent throughout its length, and tapers slightly at its caudal end (Figure S1).

#### 3.1.2 Coracoid

The sternal (ventrocaudal) end of the coracoid articulates with the coracoidal articular sulcus of the sternum at the sternal articular crest. At its omal (dorsocranial) end, it articulates with the humerus via the glenoid cavity, and the scapula at the scapular articular surface on the glenoid process. The acrocoracoid process lacks direct bony contact with the furcula, although the ventrocaudal margin of the brachial tubercle [referred to as the facies articularis clavicularis in Bertelli et al., 2014] apparently forms a syndesmosis with the omal end of the furcula. The acrocoracoid process projects craniomedially, forming a hook‐like shape that makes up the cranial portion of the triosseal canal through which the tendon of M. supracoracoideus passes. As in other Nothurinae, the procoracoid process is distinct compared to Tinaminae (Bertelli et al., 2014). Like all tinamids, our specimen has a well‐developed dorsal pneumatic foramen caudal to the scapular articular surface, which itself is shallow (Bertelli et al., 2014). The mediolateral aspect of the coracoidal body widens toward its sternal end. The coracoid's lateral process is prominent in *Nothoprocta* compared to other Nothurinae (Bertelli et al., 2014; Figure S2).

#### 3.1.3 Furcula

The furcula is U‐shaped and very thin, becoming thinner ventrally. This resembles the condition in *Rhynchotus* but contrasts with the condition in most other tinamids, where the furcula is robust throughout (Bertelli et al., 2014). Near its omal end, the furcula sits adjacent to the caudoventral margin of the brachial tubercle of the coracoid, but lacks bony contact with the coracoid, apparently forming a syndesmosis with it instead. The omal end is flared mediolaterally, and bears a blunt acromial process. A furcular apophysis is absent (Figure S3).

#### 3.1.4 Sternum

The sternum of *Nothoprocta pentlandii*, like that of other Tinamidae, is notable for its caudally elongated lateral trabeculae (Bertelli et al., 2014). The internal rostral spine, whose presence coupled with the absence of the external spine represents an osteological synapomorphy of Tinamidae (Bertelli et al., 2014), is well developed. The craniolateral processes, on the lateralmost cranial margin of the sternum, are greatly elongated in comparison with other tinamids (Bertelli et al., 2014). The sternal carina is prominent and elongated caudally, providing an enlarged area for the origins of the pectoral muscles (Mm. pectoralis et supracoracoideus). The greatly elongated carina and lateral trabeculae of the sternum are reminiscent of those of many extant phasianids (Galliformes: Phasianidae), highlighting apparent convergent morphological similarities shared by phasianids and tinamids (Houde, 1988; Figure S4).

#### 3.1.5 Humerus

The humerus of tinamids is relatively short in length. This allows for rapid movement of the wing by reducing distal inertia, and represents an adaptation to burst flight that can also be observed in several other groups of ground‐dwelling birds including phasianids (Houde, 1988). The proximal portion of the humerus is dorsoventrally expanded to form the head of the humerus. The humeral head articulates with the coracoid and scapula via the glenoid cavity, forming the glenohumeral joint. The deltopectoral crest, located on the craniodorsal margin of the proximal humerus, is well developed and extends along approximately one fifth of the length of the humerus. Adjacent to the deltopectoral crest is the dorsal tubercle, which is greatly elongated along the caudodorsal margin of the proximal humerus and hosts the tendon of M. supracoracoideus [note: this structure might more aptly be termed the supracoracoid muscular crest, but here this term is withheld due to potential ambiguity of the definition (see Baumel & Witmer, 1993)]. The bicipital crest, projecting from the ventral aspect of the proximal humerus, has a hook‐shaped profile in caudal view, an apomorphy of Nothurinae (Bertelli et al., 2014). Located just dorsodistal to the ventral tubercle is the pneumotricipital fossa, which hosts a well‐developed pneumatic foramen. The humeral shaft has a slight, laterally convex curve relative to the body in dorsal view, but is relatively straight when compared with taxa exhibiting longer humeri that take on an S‐shaped appearance when viewed in dorsal view (Houde, 1988). The brachial muscular fossa, located on the ventrocranial aspect of the distal humeral shaft, is shallow. Distally, the humerus articulates with the radius and ulna at the dorsal and ventral condyles; the former articulates with the humeral cotyle of the radius and the dorsal cotyle of the ulna, whereas the latter articulates with the ventral cotyle of the ulna. As observed in all tinamids, the ventral condyle is longer along its major axis than the dorsal condyle (Bertelli et al., 2014). Situated dorsal to the dorsal condyle and ventral to the ventral condyle are the dorsal and ventral epicondyles, respectively. On the caudal aspect, the humerotricipital groove and scapulotricipital groove incise the ventral and dorsal portions, respectively, of the distal humerus (Figure S5).

#### 3.1.6 Ulna

The ulna articulates with the ventral condyle and a portion of the dorsal condyle of the humerus, and with the ulnar articular surface of the radius at the radial incisure. At the proximal end of the ulna is the olecranon, a pointed process that hosts the insertion of M. humerotriceps. The intercotylar crest lies on the proximal ulna and corresponds with the intercondylar incisure of the distal humerus, forming a boundary between the articulations for the two condyles. On the ventral aspect just distal to the proximal end, there is a shallow fossa for the insertion of M. brachialis. The ulnar shaft has no visible remigial papillae. At its distal end, it articulates with the ulnare distally and with the radius cranially (Figure S6).

#### 3.1.7 Radius

Proximally, the radius articulates with the dorsal condyle of the humerus at the humeral cotyle, and the radial incisure of the ulna at the ulnar articular surface. The radial shaft is thinner than the ulnar shaft, and maintains roughly the same thickness throughout its length. At the distal end, it articulates with the radiale distally and with the ulna caudally (Figure S6).

#### 3.1.8 Ulnare

The ulnare is a small, ovoid bone that is the more caudally positioned of the free carpals. The crus breve articulates with the distocaudal aspect of the carpal trochlea of the ulna on its proximodorsal aspect, and with the carpal trochlea of the carpometacarpus on its distal aspect. The crus longum is relatively short and lies ventrally to the ventral rim of the carpal trochlea of the carpometacarpus. It should be noted that the crus breve and crus longum are of similar size in *Nothoprocta*, despite the implications of their names. Livezey and Zusi (2006) refer to these structures as the ramus dorsalis and ramus ventralis, respectively (Figure S7).

#### 3.1.9 Radiale

Like the ulnare, the radiale is small, and makes up the other free carpal. It is more compressed proximodistally in shape than the ulnare. It articulates directly with the distal end of the radius and the cranial portion of the carpal trochlea of the carpometacarpus (Figure S7).

#### 3.1.10 Carpometacarpus

The carpometacarpus is formed by the fused metacarpals and distal carpals. The extensor process projects from the cranial margin of the alular metacarpus. The pisiform process is a small eminence on the ventral aspect of the proximal carpometacarpus. The major metacarpus and minor metacarpus are fused at their proximal and distal ends (metacarpal symphyses), yielding an ovoid intermetacarpal space. An intermetacarpal process is absent (Figure S7).

#### 3.1.11 Manual phalanges

We follow Baumel and Witmer (1993) in referring to the three digits (I, II, and III) as the alular digit, the major digit, and the minor digit, respectively. The alular and minor digits are comprised of one phalanx each, while the major digit is composed of two. The phalanges of the major digit are approximately equal in length (Figure S7).

### 3.2 Myology

#### 3.2.1 M. latissimus dorsi pars cranialis

**FIGURE 1:**
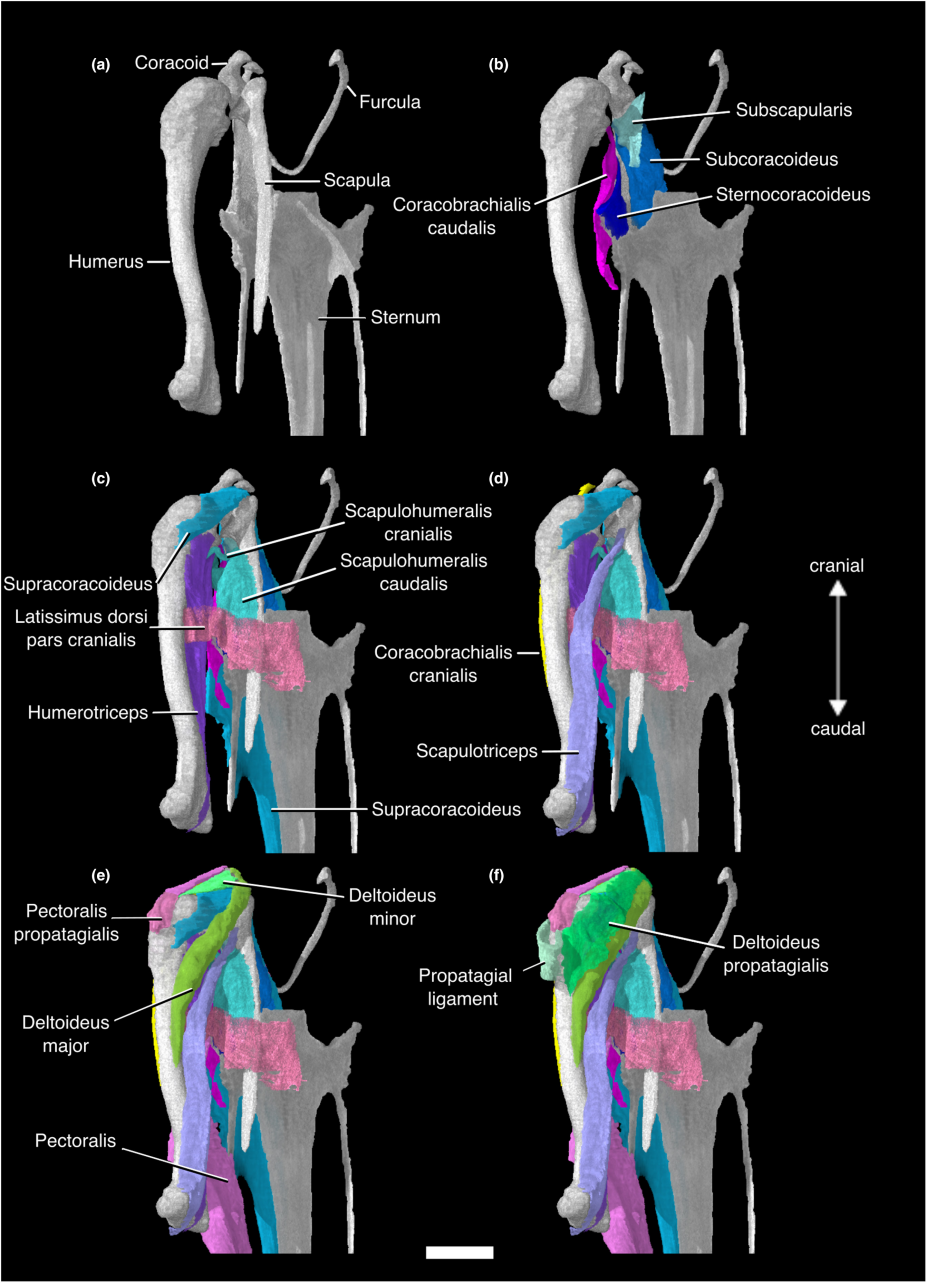
Digitally segmented muscles of the sternum, glenoid, and humerus of *Nothoprocta pentlandii*. (a) left humerus, left coracoid, left scapula, furcula, and sternum. (b) same as (a), with left scapula removed and left Mm. coracobrachialis caudalis, subscapularis, subcoracoideus, and sternocoracoideus added. (c) same as (b), with left scapula, Mm. supracoracoideus, scapulohumeralis cranialis, scapulohumeralis caudalis, latissimus dorsi pars cranialis, and humerotriceps added. (d) same as (c), with left Mm. coracobrachialis cranialis and scapulotriceps added. (e) same as (d), with left Mm. pectoralis, pectoralis pars propatagialis, deltoideus pars major, and deltoideus pars minor added. (f) same as (e), with left Mm. deltoideus propatagialis and the left propatagial ligament added. All are displayed in original articulation in dorsocaudal view. Scale bar, 10 mm.

This muscle forms a broad, dorsoventrally flattened ribbonlike structure. Its origin is on the spinal processes of the thoracic vertebrae II–VI (Suzuki et al., 2014), and it inserts on the caudal margin of the proximal shaft of the humerus (Figure 1; Figure S8; Table S1).

#### 3.2.2 M. latissimus dorsi pars caudalis

This muscle forms a large, roughly triangular sheet and makes up the superficial muscle layer of the back musculature. For the purposes of this study, we chose to illustrate only the cranial portion of this muscle. Its origin is on the spinal processes of the caudal thoracic vertebrae, thoracic vertebral ribs II–V, and the cranial margin of the ilium (Hudson et al., 1972). In our specimen, it is in contact with M. scapulohumeralis caudalis cranially. Hudson et al. (1972) reported that this muscle ends in a tendinous sheet which is attached on the skin and M. latissimus dorsi pars cranialis, M. deltoideus pars major, and M. scapulotriceps, which we believe to be the case in *Nothoprocta pentlandii*, though in our contrast‐stained specimen we were only able to locate the attachments on M. latissimus dorsi pars cranialis and M. scapulotriceps (Figure S8; Table S1).

#### 3.2.3 M. latissimus dorsi pars metapatagialis

This muscle is a long, narrow muscle superficial to M. latissimus dorsi pars caudalis. Again, in this study we chose only to illustrate the cranial portion of this muscle, in the vicinity of the flight apparatus, as we were focused on the pectoral region and this muscle became more challenging to visualize caudally. Hudson et al. (1972) found this muscle's origin to be on the spinal processes of the caudal thoracic vertebrae, and the insertion on M. serratus superficialis metapatagialis (Figure S8; Table S1).

#### 3.2.4 M. supracoracoideus

**FIGURE 2:**
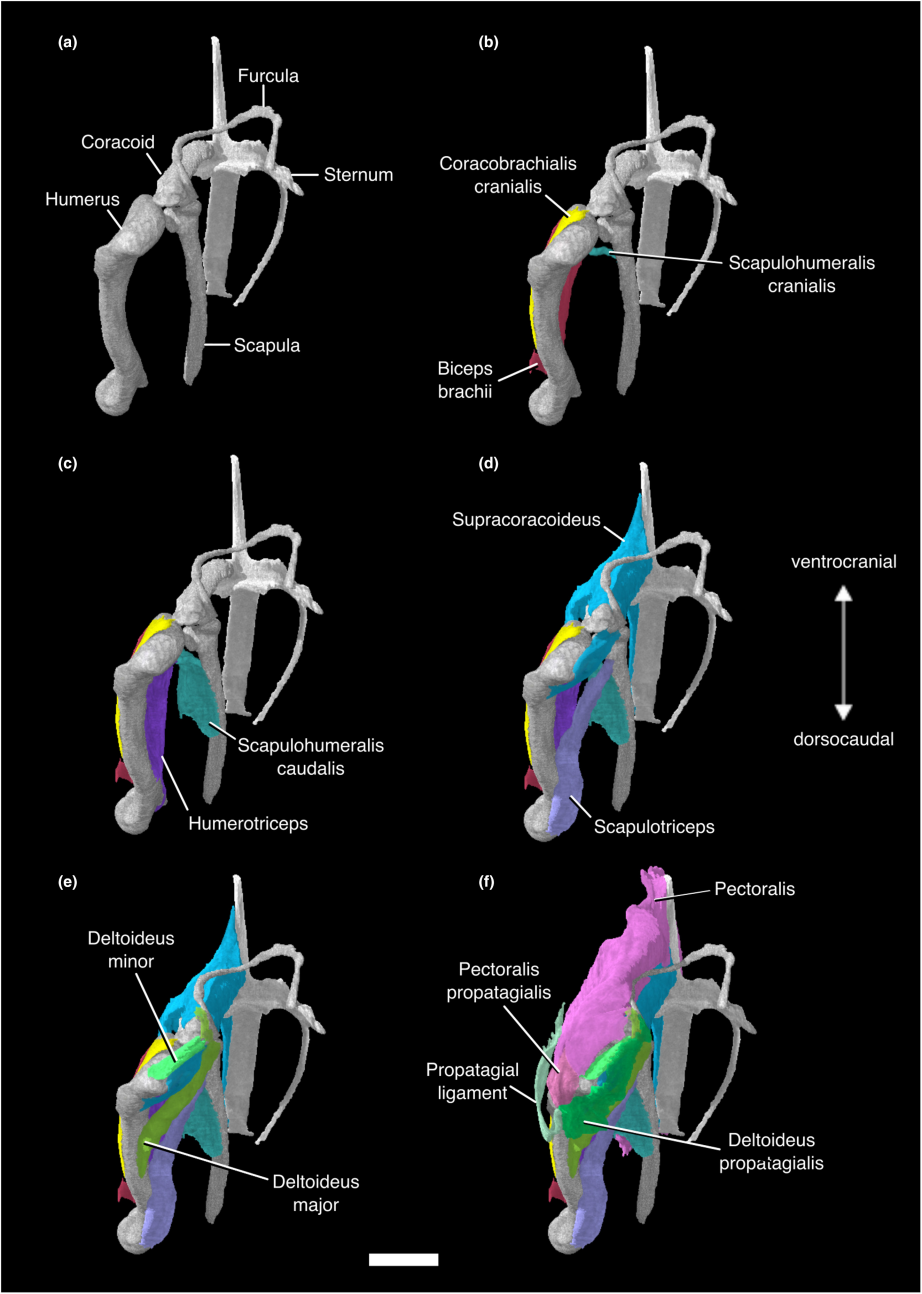
Digitally segmented muscles of the sternum, glenoid, and humerus of *Nothoprocta pentlandii*. (a) left humerus, coracoid, scapula, furcula, and sternum. (b) same as (a), with left Mm. coracobrachialis cranialis, biceps brachii, and scapulohumeralis cranialis added. (c) same as (b), with left Mm. humerotriceps and scapulohumeralis caudalis added. (d) same as (c), with left Mm. supracoracoideus and scapulotriceps added. (e) same as (d), with left Mm. deltoideus pars major and deltoideus pars minor added. (f) same as (e), with left Mm. pectoralis, pectoralis pars propatagialis, deltoideus pars propatagialis, and the propatagial ligament added. All are displayed in original articulation in dorsocranial view. Scale bar, 10 mm.

**FIGURE 3:**
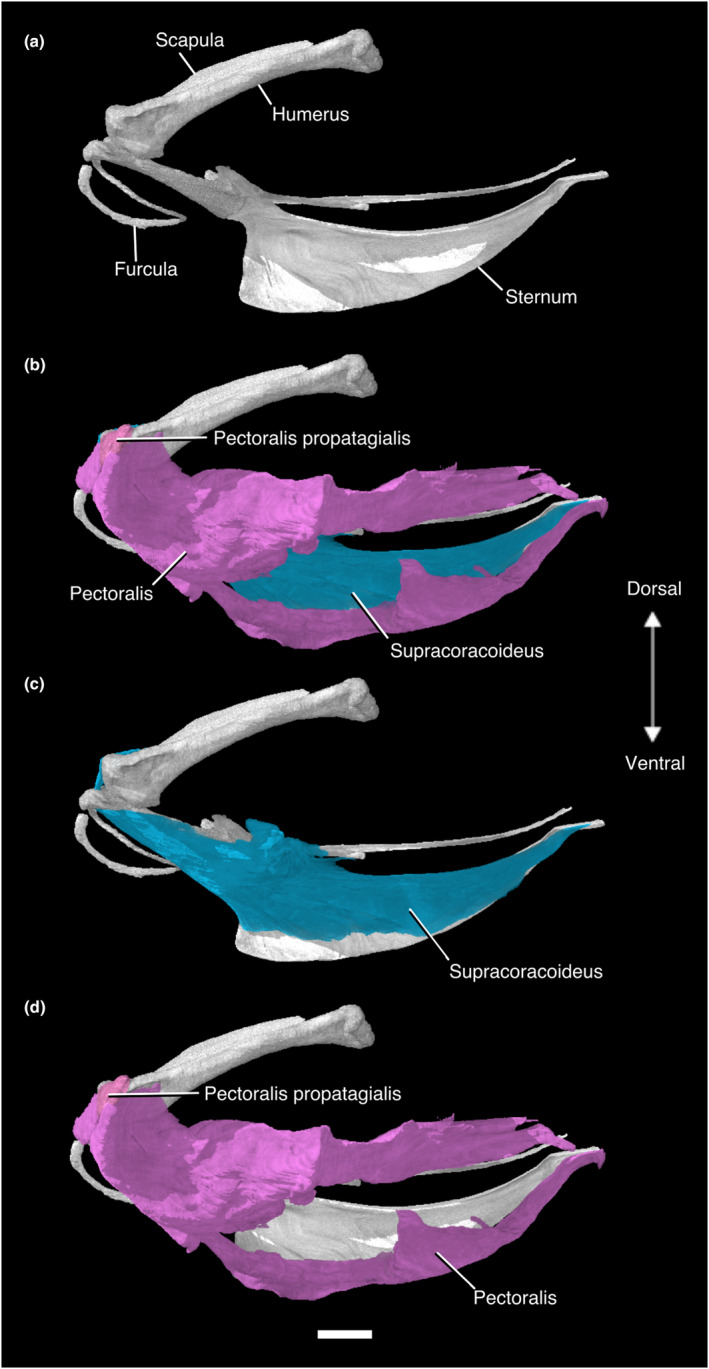
Digitally segmented pectoral flight muscles of *Nothoprocta pentlandii*. (a) sternum, furcula, left coracoid, left humerus, left scapula. (b) same as (a), with left Mm. supracoracoideus, pectoralis, and pectoralis pars propatagialis added. (c) same as (a), with left M. supracoracoideus added. (d) same as (a), with left Mm. pectoralis and pectoralis pars propatagialis added. All are displayed in original articulation in left lateral view. Scale bar, 10 mm.

**FIGURE 4:**
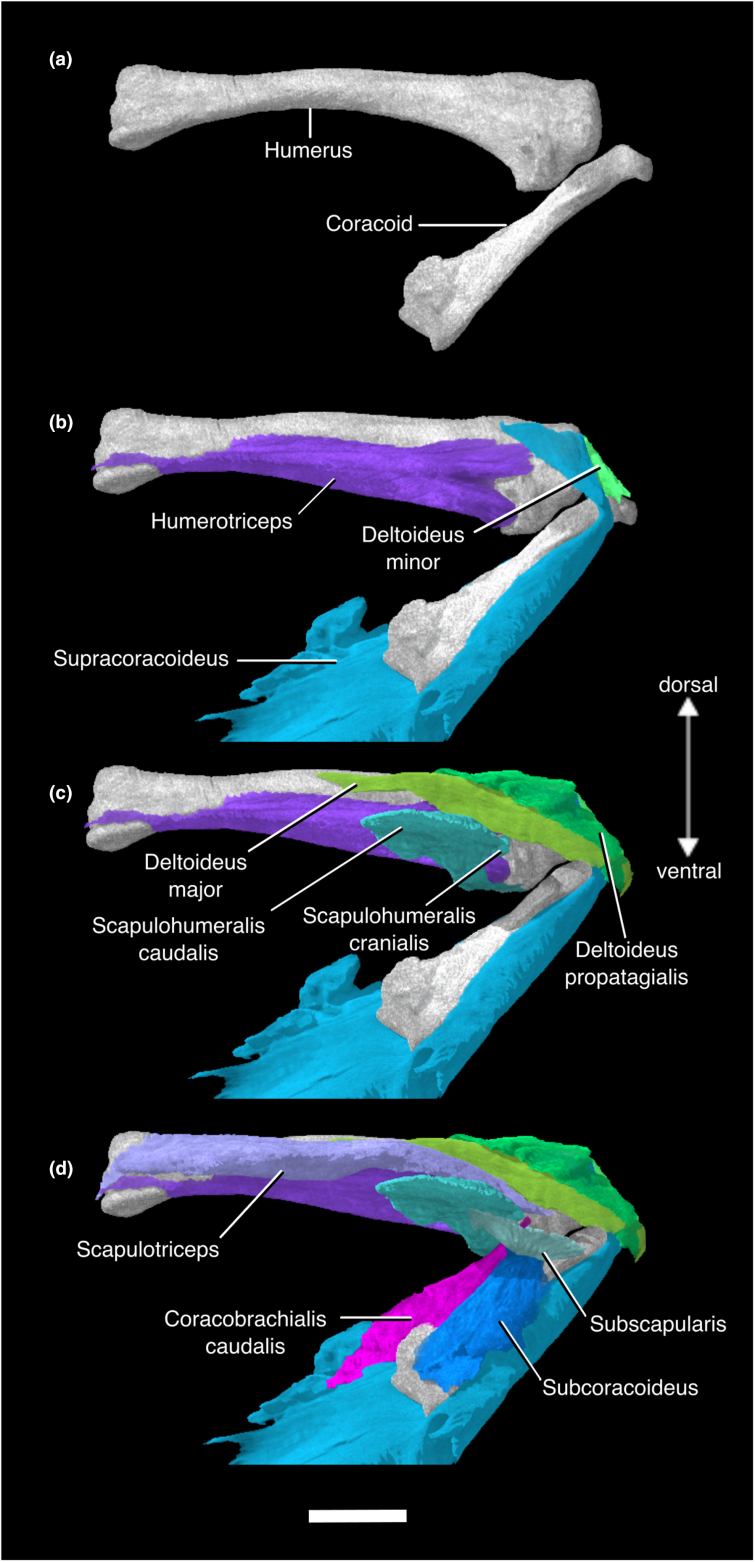
Digitally segmented muscles of the caudal humerus of *Nothoprocta pentlandii*. (a) left humerus and coracoid. (b) same as (a), with left Mm. humerotriceps, deltoideus pars minor, and supracoracoideus added. (c) same as (b), with left M. deltoideus pars major, deltoideus pars propatagialis, scapulohumeralis cranialis, and scapulohumeralis caudalis added. (d) same as (c), with left Mm. scapulotriceps, coracobrachialis caudalis, subscapularis, and subcoracoideus added. All are displayed in original articulation in medial view. Scale bar, 10 mm.

This muscle lies deep to M. pectoralis. The massive, fleshy origin of this muscle lies on the sternal carina, the ventral surface of the sternal notch membrane, and the ventromedial aspect of the coracoidal body. Its origin on the sternal carina stretches back to the caudalmost portion of the sternum but does not reach the cranial two thirds of the ventralmost portion of the carina. M. supracoracoideus passes through the triosseal canal as a thick tendon and has a tendinous insertion on the dorsal tubercle of the proximal humerus, caudal to the insertion of M. deltoideus pars minor (Figures 1, 2, 3, 4; Figure S9; Table S1).

#### 3.2.5 M. pectoralis

**FIGURE 5:**
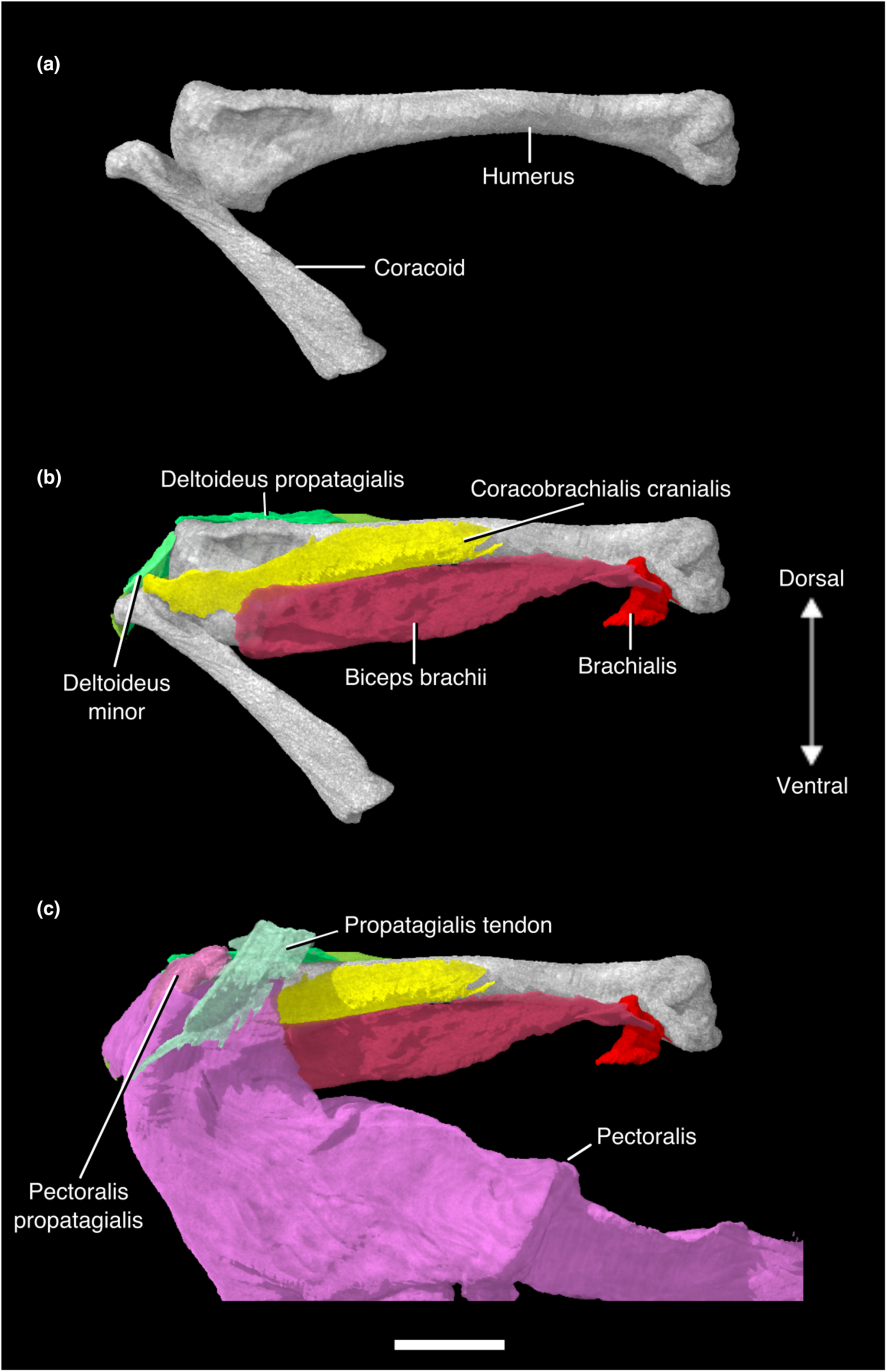
Digitally segmented muscles of the cranial humerus of *Nothoprocta pentlandii*. (a) left coracoid and humerus. (b) same as (a), with left Mm. deltoideus pars minor, deltoideus pars propatagialis, coracobrachialis cranialis, biceps brachii, and brachialis added. (c) same as (b), with left Mm. pectoralis, pectoralis pars propatagialis, and the left propatagial ligament added. All are displayed in original articulation in lateral view. Scale bar, 10 mm.

This massive muscle has a fleshy origin on the ventral portion of the sternal carina, the sternal notch membrane, the ventrolateral surface of the furcula, and the lateral trabeculae of the sternum. It has a broad, tendinous insertion on the cranioventral aspect of the deltopectoral crest of the humerus. The muscle can be divided into costobrachial and sternobrachial parts that originate on the lateral trabecula and ventral portion of the sternal carina, respectively. The intermuscular aponeurosis was not visible in our contrast‐stained specimen (Figures 1, 2, 3 and 5; Figure S9; Table S1).

#### 3.2.6 M. pectoralis pars propatagialis

This muscle branches off the main belly of M. pectoralis near its insertion on the deltopectoral crest as a robust, fleshy slip. It passes dorsally to the deltopectoral crest and appears to insert on the cranioventral margin of the proximal portion of the propatagial ligament and on M. deltoideus pars propatagialis (Figures 1, 2, 3 and 5; Figure S9; Table S1).

#### 3.2.7 M. sternocoracoideus

M. sternocoracoideus is short and wide mediolaterally. Its fleshy origin is on the dorsal and dorsomedial surfaces of the craniolateral process of the sternum. It has a broad, fleshy insertion on the dorsal surface of the lateral process of the coracoid (Figure 1; Table S1).

#### 3.2.8 M. scapulohumeralis cranialis

M. scapulohumeralis cranialis forms a thin cord between the scapula and humerus, and has a fleshy origin and insertion. The origin is on the lateral surface of the scapular neck cranial to the origin of the lateral head of M. subscapularis, and it inserts on the proximal humerus on the distal edge of the pneumatic foramen (Figures 1, 2 and 4; Table S1).

#### 3.2.9 M. scapulohumeralis caudalis

M. scapulohumeralis caudalis is substantially larger than M. scapulohumeralis cranialis. The fleshy origin of this muscle extends along the lateral surface of the scapula, from the caudal margin of the origin of M. scapulohumeralis cranialis to the caudal end of the scapula. Its thick tendon of insertion passes ventrally to M. scapulohumeralis cranialis, and has a wide insertion that stretches across the ventral margin of the proximal bicipital crest (Figures 1, 2 and 4; Table S1).

#### 3.2.10 M. subscapularis

M. subscapularis has a wide, fleshy origin on the lateral and medial surfaces of the cranial scapula. These origins are sometimes referred to as the caput laterale and mediale (Suzuki et al., 2014; Vanden Berge & Zweers, 1993). The caput laterale is bordered by the origins of M. scapulohumeralis cranialis and M. scapulohumeralis caudalis caudally. The medial head is much larger than the lateral one. It inserts via a shared tendon with M. subcoracoideus onto the proximal aspect of the ventral tubercle of the humerus (Figures 1 and 4; Table S1).

#### 3.2.11 M. subcoracoideus

This muscle has a broad, fleshy origin on the dorsolateral aspect of the internal rostral spine of the sternum and the dorsomedial aspect of the sternocoracoclaviclar membrane. This muscle runs dorsally to the coracoid without contacting the bone itself, and merges with the caudoventral surface of M. subscapularis. The two muscles then form a common tendon of insertion onto the ventral tubercle of the humerus (Figures 1 and 4; Table S1).

#### 3.2.12 M. deltoideus pars major

This relatively large muscle appears to have a fleshy origin on the medial aspect of the acrocoracoid process of the coracoid. The origin may extend onto the connective tissues surrounding the triosseal canal, but this could not be visualized in our scans. The muscle passes dorsally to the cranial scapula and inserts on the dorsocaudal surface of the proximal humeral shaft. The insertion appears to be fleshy, although Hudson et al. (1972) stated that the insertion was via a tendinous sheet (Figures 1, 2 and 4; Table S1).

#### 3.2.13 M. deltoideus pars minor

M. deltoideus pars minor has a fleshy origin on the lateral aspect of the acrocoracoid process of the coracoid, and the acromial process of the furcula ventral and lateral to that of M. deltoideus pars propatagialis. It has a short muscle body, and inserts onto the proximodorsal margin of the humeral head. The presence of the caput ventrale (or the “long head”) mentioned by Hudson et al. (1972) could not be confirmed in our scan. It is absent in all other non‐tinamid palaeognaths examined by previous authors (McGowan, 1982; Suzuki et al., 2014) (Figures 1, 2, 4 and 5; Table S1).

#### 3.2.14 M. deltoideus pars propatagialis

The muscle forms a broad, thin ribbon superficial to M. deltoideus pars major and minor. The fleshy origin of this muscle lies on the dorsomedial aspect of the acromial process of the furcula. This muscle passes over the craniodorsal aspect of the proximal humerus where it becomes the propatagial ligament (Figures 1, 2, 4 and 5; Table S1).

#### 3.2.15 Propatagial ligaments

The propatagial ligaments are divided into propatagial and elbow‐limiting ligaments (propatagialis longus and brevis tendons, respectively, in Hudson et al., 1972). The propatagial ligament is a long sheet of connective tissue spanning the leading edge of the wing from the shoulder joint to the wrist joint. Its cranio‐caudal aspect narrows distally, and it is dorsoventrally narrow throughout. The elbow‐limiting ligament forms a broad sheet that is tendinous throughout (Hudson et al., 1972), and inserts on the distal belly of M. extensor carpi radialis. In our specimen, only the proximal portion of the elbow‐limiting ligament could be visualized, and the insertion was not observable (Figures 1, 2 and 5; Table S1).

#### 3.2.16 M. coracobrachialis cranialis

M. coracobrachialis cranialis has a tendinous origin on the acrocoracoid process of the coracoid. Hudson et al. (1972) and Suzuki et al. (2014) reported that its origin was located near that of M. biceps brachii in their specimens, but we cannot confirm this as we were unable to locate the tendon of origin for M. biceps brachii. The fleshy insertion of M. coracobrachialis cranialis occupies a large portion of the dorsocranial surface of the humerus distal to the insertion of M. pectoralis and the humeral attachment of M. biceps brachii, ventral to the deltopectoral crest. It contacts M. humerotriceps and M. biceps brachii ventrally along the majority of its insertion (Figures 1, 2 and 5; Table S1).

#### 3.2.17 M. coracobrachialis caudalis

This muscle is relatively large, and originates on the ventral surface of the craniolateral process of the sternum, the ventrolateral surface of the lateral process of the coracoid, and the fascia of M. supracoracoideus. It inserts on the dorsal aspect of the ventral tubercle of the humerus, just proximal to the pneumatic foramen (Figures 1 and 4; Table S1).

#### 3.2.18 M. scapulotriceps

This muscle has a fleshy origin on the lateral surface of the cranial scapula. We were only able to observe one tendon of origin, but Hudson et al. (1972) and Suzuki et al. (2014) described two tendinous slips originating on the lateral surface, one caudal to the humeral articular surface, the other cranial to the origin of M. scapulohumeralis cranialis. Hudson et al. (1972) also mentioned that additional fleshy attachment on the scapula is variably present among tinamid genera including *Nothoprocta*, and there is a tendinous attachment on the humerus associated with M. latissimus dorsi pars caudalis. This muscle forms a fleshy belly that runs parallel to the caudodorsal margin of the humerus. It becomes tendinous as it passes over the scapulotricipital groove of the humerus and inserts on the olecranon of the ulna (Figures 1, 2 and 4; Table S1).

#### 3.2.19 M. humerotriceps

M. humerotriceps has a broad origin on the humeral shaft. There is a prominent notch in the origin due to the presence of the pneumatic foramen and the insertion of M. scapulohumeralis cranialis. M. humerotriceps is subdivided into a large central and ventral belly, along with a smaller dorsal belly that originates on the proximal caudal margin before merging with the central belly. The central and ventral bellies converge distally as the muscle narrows and turns into a tendon near its insertion. In our specimen, the tendon of insertion becomes invisible as it passes over the humerotricipital groove of the humerus. Suzuki et al. (2014) confirmed that it inserts onto the olecranon in *Eudromia elegans*, which we believe holds true in *Nothoprocta pentlandii* (Figures 1, 2 and 4; Table S1).

#### 3.2.20 M. biceps brachii

This muscle lies ventral to the belly of M. coracobrachialis cranialis, cranial to that of M. humerotriceps, and deep to the distal tendon of M. pectoralis. The tendon of origin for this muscle could not be visualized. Hudson et al. (1972) and Suzuki et al. (2014) reported two origins for this muscle in their specimens: one on the craniolateral aspect of the omal end of the coracoid, and the other on the bicipital crest of the humerus. In our specimen, M. biceps brachii can be observed from its origin at the bicipital crest, running parallel to the cranial surface of the humerus and tapering slightly to its tendon of insertion. The tendon of insertion is thick, and enters the ventral side of the elbow joint, inserting on the caudal and cranial aspects of the proximal ends of the radius and ulna, respectively (Figures 2 and 5; Figure S10; Table S1).

#### 3.2.21 M. expansor secundariorum

This muscle was poorly visualized in our specimen. Hudson et al. (1972) reported its origin as the distal portion of M. latissimus dorsi pars caudalis, the ventral edge of M. scapulohumeralis caudalis, and the caudolateral edge of M. subcoracoideus. Here, only the origin on M. scapulohumeralis caudalis could be visualized. M. expansor secundariorum runs parallel to the caudal aspect of M. humerotriceps, though only small segments of this portion of the muscle could be observed. It becomes fleshy near its insertion on the quills of the proximal three secondary remiges (Suzuki et al., 2014; Figure S10; Table S1).

#### 3.2.22 M. brachialis

M. brachialis forms a short, fleshy band between the ventral humerus and ulna. Its origin is on the brachial muscle fossa on the cranioventral aspect of the distal humeral shaft and it inserts onto the brachial muscle impression of the ventral surface of the proximal ulna, which is developed into a fossa (Figure 5; Figure S10; Table S1).

#### 3.2.23 M. pronator superficialis

**FIGURE 6:**
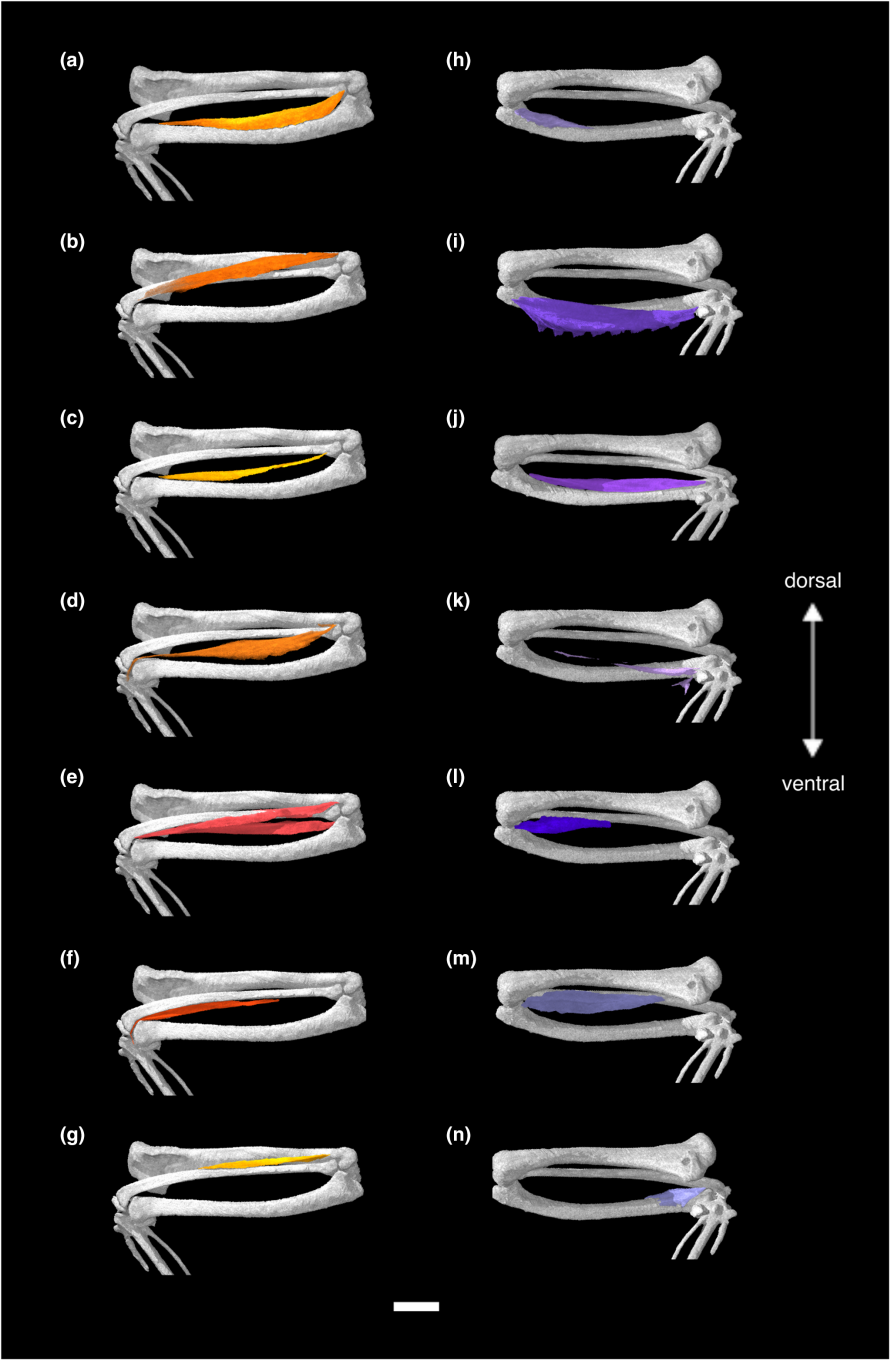
Digitally segmented forearm muscles of *Nothoprocta pentlandii*, displayed individually. (a) through (g) show the left humerus, radius, ulna, radiale, ulnare, and carpometacarpus in original articulation in lateral view. H through N show the left humerus, radius, ulna, radiale, ulnare, and carpometacarpus in original articulation in medial view. (a) left M. ectepicondylo‐ulnaris. (b): left M. extensor carpi radialis. (c) left M. extensor carpi ulnaris. (d) left M. extensor digitorum communis. (e): left M. extensor longus alulae. (f) left M. extensor longus digiti majoris. (g) left M. supinator. (h) left M. entepicondylo‐ulnaris. (i) left M. flexor carpi ulnaris. (j) left M. flexor digitorum profundus. (k): left M. flexor digitorum superficialis. (l) left M. pronator profundus. (m) left pronator superficialis. (n) left M. ulnometacarpalis ventralis. Note that the orientations in this figure refer to those in the flexed wing rather than the standard anatomical orientations for the extended wing. Scale bar, 10 mm.

**FIGURE 7:**
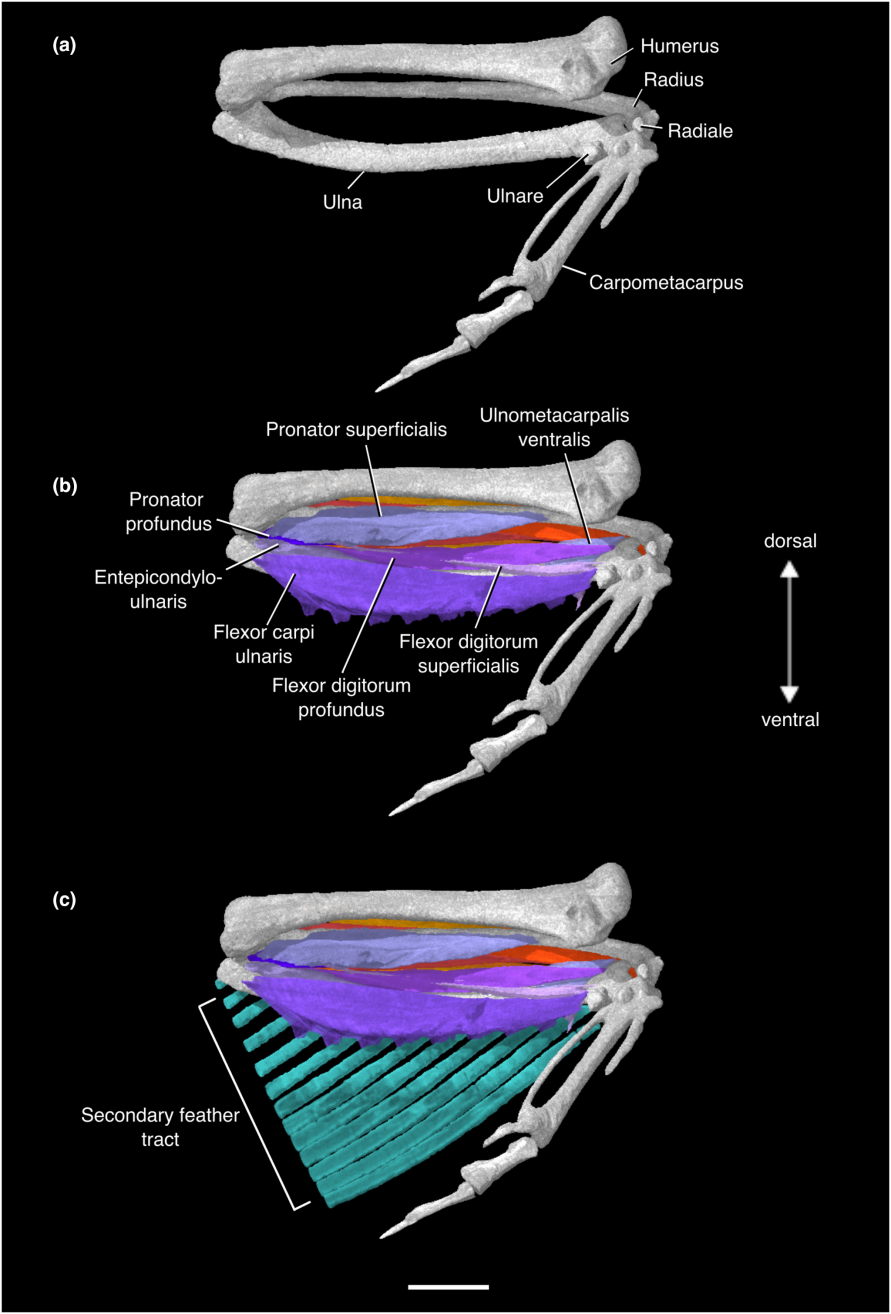
Digitally segmented ventral forearm of *Nothoprocta pentlandii*. (a) Left humerus, radius, ulna, radiale, ulnare, carpometacarpus, and phalanges. (b) same as (a), with left Mm. entepicondylo‐ulnaris, flexor carpi ulnaris, flexor digitorum profundus, flexor digitorum superficialis, pronator profundus, pronator superficialis, and ulnometacarpalis ventralis added. (c) same as (b), with the secondary feather tract added. Mm. supinator, extensor longus alulae, and extensor carpi ulnaris are unlabelled, see Figure 8. All are displayed in original articulation in medial view. Note that the orientations in this figure refer to those in the flexed wing rather than the standard anatomical orientations for the extended wing. Scale bar, 10 mm.

The tendon of origin was unobservable proximal to the ventral epicondyle of the humerus; Hudson et al. (1972) and Suzuki et al. (2014) confirmed the ventral epicondyle as the origin in their specimens. It runs along the cranioventral surface of the radius, inserting on the distal third of the radial shaft (Figures 6 and 7; Table S1).

#### 3.2.24 M. pronator profundus

M. pronator profundus is the smaller of the two pronators. This muscle is deep to M. pronator superficialis, and also has an origin on the ventral epicondyle of the humerus distal to that of M. pronator superficialis (Hudson et al., 1972; Suzuki et al., 2014). It inserts midway down the radial shaft, on the caudal margin of the bone. This muscle is notably absent in ostriches (Suzuki et al., 2014), emus (Maxwell & Larsson, 2007), kiwi (McGowan, 1982), and possibly rheas (Lo Coco et al., 2022; Suzuki et al., 2014), and Beddard (1898) reported that only a single pronator is present in ratites (Figures 6 and 7; Table S1).

#### 3.2.25 M. entepicondylo‐ulnaris

This muscle's tendon of origin is not visible. Hudson et al. (1972) and Suzuki et al. (2014) reported the tendon being fused with the tendon of M. pronator profundus. This muscle is fleshy near its origin and tapers near its insertion approximately one third of the way down the ventral ulnar shaft. Notably, this muscle is found only in palaeognaths and galloanserans (Livezey & Zusi, 2006; Vanden Berge & Zweers, 1993), indicating that it may have been present in the last common ancestor of crown birds, and lost in stem Neoaves (Figures 6 and 7; Table S1).

#### 3.2.26 M. flexor digitorum superficialis

This muscle is said to arise from a thick superficial fascia in the ventral forearm (Hudson et al., 1972; Vanden Berge & Zweers, 1993), which is not visible here. Suzuki et al. (2014) interpreted this fascia as a thin, tendinous origin of this muscle, arising distal to the origin of M. pronator profundus on the ventral epicondyle of the humerus in *Eudromia elegans*. In our specimen, the belly of this muscle can be visualized between the proximal ulnar shaft and the ulnare. It is deep to M. flexor carpi ulnaris and superficial to M. flexor digitorum profundus in the forearm. According to Hudson et al. (1972) and Suzuki et al. (2014), the muscle becomes tendinous near the wrist joint, passes through a groove on the ventral aspect of the ulnare, and inserts on the ventral surface of the distal phalanx of the major digit. We interpret the insertion of this muscle on the ulnare mentioned by Suzuki et al. (2014) as that of the fascia mentioned above (as per Hudson et al., 1972). Here, this muscle becomes invisible past the ventral aspect of the ulnare (Figures 6 and 7; Table S1).

#### 3.2.27 M. flexor digitorum profundus

This muscle's origin is on the ventral margin of the ulnar shaft. It runs the length of the ulnar shaft, tapering as it reaches the distal end. It forms the deepest layer of ventral forearm musculature along with M. ulnometacarpalis ventralis. Distal to the wrist joint, the M. flexor digitorum profundus can no longer be visualized. Hudson et al. (1972) and Suzuki et al. (2014) found that it became tendinous near the wrist joint, with the tendon passing through a groove cranial to the pisiform process of the carpometacarpus. The tendon divided, with one branch inserting on the alular phalanx, and the other fusing or enclosed with the tendon of M. flexor digitorum superficialis to insert on the ventral surface of the distal phalanx of the major digit (Hudson et al., 1972; Suzuki et al., 2014; Figures 6 and 7; Table S1).

#### 3.2.28 M. flexor carpi ulnaris

This large muscle runs along the ventrocaudal aspect of the ulnar shaft, caudal to M. flexor digitorum superficialis and M. entepicondylo‐ulnaris. We could not locate the tendon of origin. Hudson et al. (1972) and Suzuki et al. (2014) reported that it originates on the distal aspect of the ventral epicondyle of the humerus in *Eudromia elegans*. It inserts via a thick tendon onto the ventrocaudal aspect of the ulnare. There are eight clear connection points for the bases of secondary remiges along the muscle's caudal margin, which were referred to M. flexor carpi ulnaris pars remigalis by Vanden Berge and Zweers (1993) (Figures 6 and 7; Table S1).

#### 3.2.29 M. ulnometacarpalis ventralis

M. ulnometacarpalis ventralis has a fleshy origin on the ventral surface of the distal ulna. It can no longer be found distal to the wrist joint. Suzuki et al. (2014) and Hudson et al. (1972) found that it became tendinous as it passed ventrally to the wrist joint before turning around the cranial aspect of the radiale and inserting onto the craniodorsal surface of the proximal end of the carpometacarpus (Figures 6 and 7; Table S1).

#### 3.2.30 M. extensor carpi radialis

**FIGURE 8:**
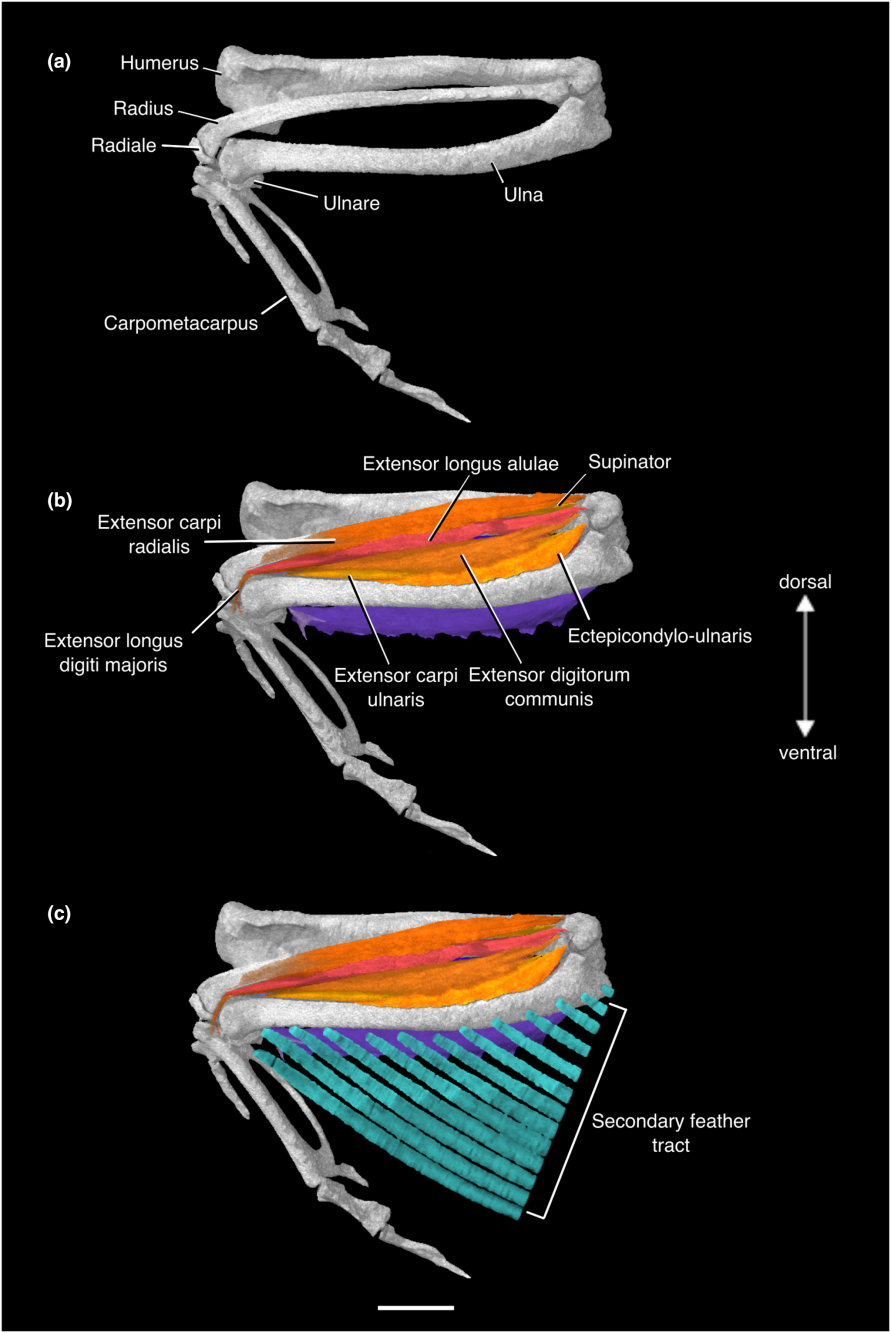
Digitally segmented dorsal forearm of *Nothoprocta pentlandii*. A: Left humerus, radius, ulna, radiale, ulnare, carpometacarpus, and phalanges. B: same as A, with left Mm. ectepicondylo‐ulnaris, extensor carpi radialis, extensor carpi ulnaris, extensor digitorum communis, extensor longus alulae, extensor longus digiti majoris, and supinator added. C: same as B, with the secondary feather tract added. Left Mm. flexor carpi ulnaris and flexor digitorum superficialis are unlabelled, see Figure 7. All are displayed in original articulation in lateral view. Note that the orientations in this figure refer to those in the flexed wing rather than the standard anatomical orientations for the extended wing. Scale bar, 10 mm.

The origin of this muscle is on the proximal portion of the dorsal epicondyle of the humerus, dorsal to that of M. supinator. It is large and fleshy throughout most of its length, and lies dorsal to M. extensor longus alulae. It abruptly narrows and turns into a thin tendon that runs parallel to the tendon of M. extensor longus alulae as it reaches the distal end of the radius. Distal to this point, it is unobservable. Suzuki et al. (2014) reported the insertion as the cranioproximal aspect of the alular metacarpus in *Eudromia elegans*, cranial to the insertion of M. extensor longus alulae. Hudson et al. (1972) reported that it fuses with the tendon of M. extensor longus alulae just prior to insertion (Figures 6 and 8; Table S1).

#### 3.2.31 M. supinator

This muscle has a tendinous origin on the dorsal epicondyle of the humerus. It is narrow throughout its length, and has a fleshy insertion midway down the craniodorsal aspect of the radial shaft. It is deep to M. extensor carpi radialis and contacts the radial origin of M. extensor longus alulae caudally (Figures 6 and 8; Table S1).

#### 3.2.32 M. extensor digitorum communis

This muscle has a long tendon of origin that originates on the dorsal epicondyle of the humerus. Suzuki et al. (2014) reported a second, smaller tendon of origin on the proximal end of the ulna distal to their “intercondylar crest”, but we were unable to locate this tendon. Suzuki et al. (2014) reported the tendon of origin on the dorsal epicondyle of the humerus as distal to the origins of M. supinator and M. extensor carpi radialis, but in our specimen it appears to be positioned proximally to those origins. Hudson et al. (1972) found the origin proximal to that of M. supinator, but distal to that of M. extensor carpi radialis. This muscle tapers and becomes a thin tendon distally, where it passes through a retinaculum on the dorsal aspect of the wrist joint. We can no longer observe this muscle distal to the proximal portion of the dorsal surface of the alular metacarpus. Hudson et al. (1972) and Suzuki et al. (2014) reported that the tendon of insertion divides, inserting onto both the dorsocaudal side of the alula near the base of the digit and the base of the first phalanx of the major digit, and a separate insertion is present on the distal phalanx of the major digit (Figures 6 and 8; Table S1).

#### 3.2.33 M. ectepicondylo‐ulnaris

We were unable to locate the origin of this muscle, although Hudson et al. (1972) and Suzuki et al. (2014) reported the origin as a common tendon with M. extensor carpi ulnaris which arises from the dorsal epicondyle of the humerus. The belly of this muscle stretches across the interosseous space between the radius and ulna. It inserts on the craniodorsal surface of the distal midshaft of the ulna (Figures 6 and 8; Table S1).

#### 3.2.34 M. extensor carpi ulnaris

This forearm muscle forms a thin strip and appears to originate on the fascia of M. ectepicondylo‐ulnaris, whereas it has been said to have a common tendon with M. ectepicondylo‐ulnaris (Hudson et al., 1972; Suzuki et al., 2014). In our specimen, the insertion is not visible as the muscle can no longer be observed just proximal to the distal end of the ulna. Hudson et al. (1972) and Suzuki et al. (2014) reported that it becomes tendinous near the wrist joint in their specimens, and follows the tendon of M. extensor digitorum communis before inserting onto the caudal margin of the major metacarpus (Figures 6 and 8; Table S1).

#### 3.2.35 M. extensor longus alulae

This muscle has two fleshy points of origin; one on the dorsal surface of the proximal ulna distal to the radial articular surface and the other covering the caudal margin of the proximal radial shaft. We were unable to observe it distal to the wrist joint, but Suzuki et al. (2014) stated that the tendon of insertion extended to the proximodorsal surface of the alular metacarpus in *Eudromia elegans*, ventral to the insertion of M. extensor carpi radialis, whereas Hudson et al. (1972) stated that the tendons of these muscles fused with each other (Figures 6 and 8; Table S1).

#### 3.2.36 M. extensor longus digiti majoris

This muscle has a proximodistally long origin on the caudoventral margin of the distal two‐thirds of the radius. It runs across the craniodistal rim of the dorsal condyle of the ulna before becoming tendinous. We could not observe the tendon distal to the proximal portion of the major metacarpus. Hudson et al. (1972) and Suzuki et al. (2014) described its tendinous insertion on the first phalanx of the major digit in their specimens [although Suzuki et al., 2014 (Figure 7) illustrated an insertion on the second phalanx, contradicting their descriptions] (Figures 6 and 8; Table S1).

#### 3.2.37 M. ulnometacarpalis dorsalis

**FIGURE 9:**
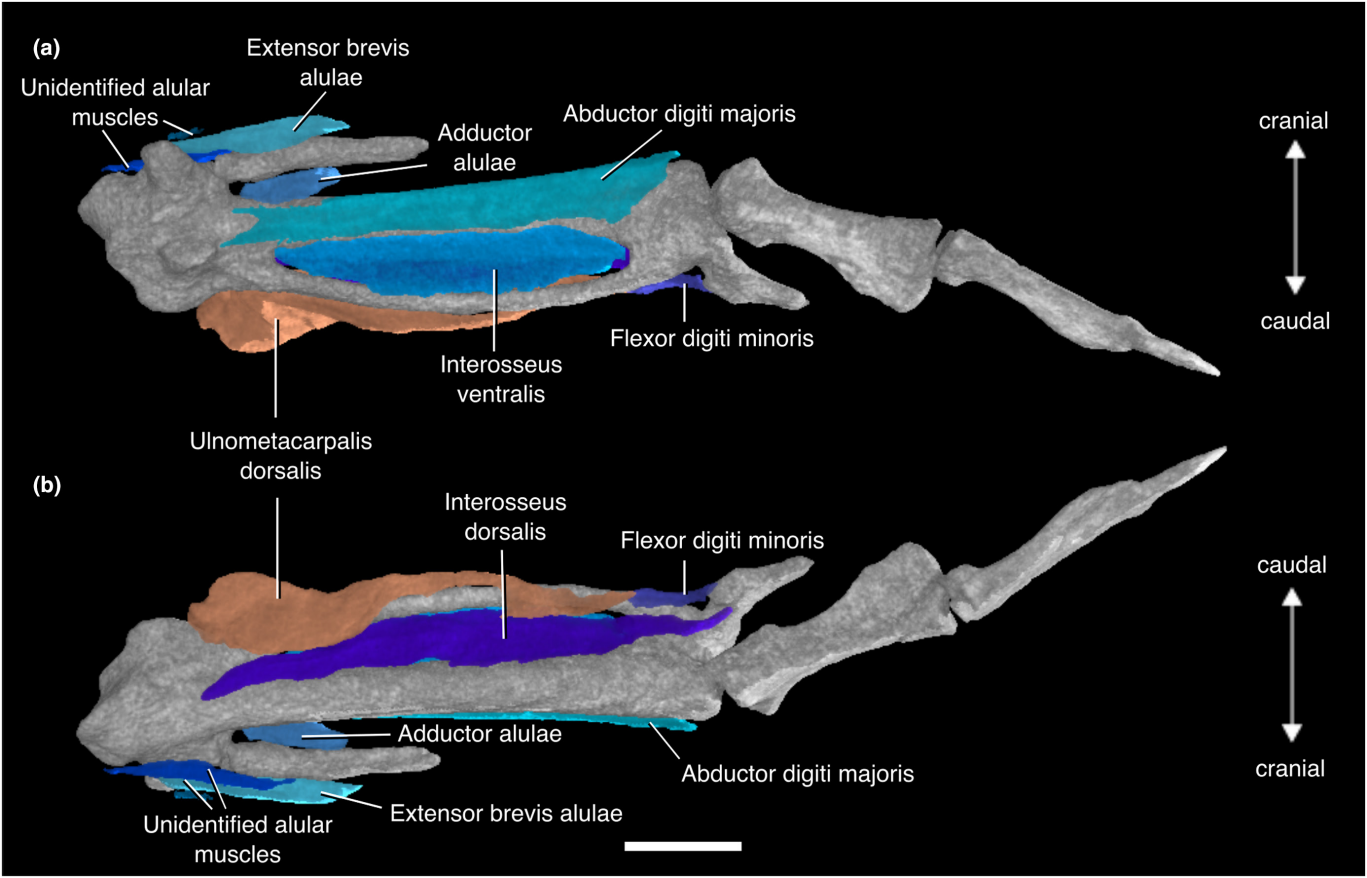
Digitally segmented muscles of the manus of *Nothoprocta pentlandii*. A: left radiale, ulnare, carpometacarpus, and phalanges in original articulation, with left Mm. extensor brevis alulae, adductor alulae, ulnometacarpalis dorsalis, interosseus ventralis, abductor digiti majoris, and flexor digiti minoris, in ventral view. B: same osteology, with left Mm. extensor brevis alulae, adductor alulae, ulnometacarpalis dorsalis, interosseus dorsalis, abductor digiti majoris, and flexor digiti minoris, in dorsal view. Scale bar, 5 mm. Mm. abductor alulae and flexor alulae could not be identified with confidence.

We found the origin of this muscle on the caudal margin of the distalmost ulnar shaft and the dorsal surface of the ulnare. Hudson et al. (1972) found the origin to be the craniodorsal margin of the distal ulna. It inserts as a long strip along the caudal surface of the minor metacarpus (Figure 9; Table S1).

#### 3.2.38 M. abductor alulae

In our specimen, we could not identify this muscle with confidence. Hudson et al. (1972) reported that it has a fleshy origin stemming from the ventral surface of the distal tendon of M. extensor carpi radialis, and inserts on the antero‐ventral surface of the alular phalanx. Suzuki et al. (2014) described the origin as being near the insertion of M. extensor carpi radialis, and the insertion on the lateral surface of the alular phalanx (Table S1).

#### 3.2.39 M. flexor alulae

This muscle was poorly visualized in our specimen, likely due to its small size. Hudson et al. (1972) found the origin on the ventral carpometacarpus, between the ankylosed first metacarpal and the pisiform process, while Suzuki et al. (2014) simply described it as being on the proximal ventral carpometacarpus. Hudson et al. (1972) reported the fleshy insertion to be on the postero‐ventral edge of the alular phalanx. The insertion was reported as the caudal margin of the ventral surface of the alular phalanx by Suzuki et al. (2014) (Table S1).

#### 3.2.40 M. adductor alulae

This muscle's origin is on the cranial margin of the proximal shaft of the major metacarpus. The muscle is fleshy throughout. It inserts onto the caudal margin of the alular phalanx. Hudson et al. (1972) and Suzuki et al. (2014) reported that it also inserts onto the two quills attached to the alula in *Eudromia elegans* (Figure 9; Table S1).

#### 3.2.41 M. extensor brevis alulae

The origin of this muscle is on the proximal portion of the dorsal surface of the alular metacarpus, although Hudson et al. (1972) mentioned its origin from the common tendon of M. extensor carpi radialis and M. extensor longus alulae. Like M. abductor alulae, it follows the cranial margin of the alula. It inserts on the cranial margin of the alular phalanx, proximal to the insertion of M. abductor alulae. Hudson et al. (1972) found the extent of the insertion site to be variable among tinamid genera (Figure 9; Table S1).

#### 3.2.42 M. abductor digiti majoris

This muscle has a long, fleshy origin on the ventral surface of the major metacarpus. We were unable to locate the insertion, which Hudson et al. (1972) and Suzuki et al. (2014) reported to be on the cranial margin of the proximal end of the first phalanx of the major digit in *Eudromia elegans* (Figure 9; Table S1).

#### 3.2.43 M. interosseus dorsalis

The origin of M. interosseus dorsalis lies on the caudodorsal margin of the major metacarpus across to the craniodorsal margin of the minor metacarpus. It fills the intermetacarpal space between the major and minor metacarpi. Distally, it narrows and turns into a thin tendon, and then appears to insert onto the proximal portion of the dorsal surface of the phalanx of the minor digit, although the actual insertion cannot be visualized. This interpretation matches what was described by Suzuki et al. (2014), who also reported an insertion on the phalanx of the minor digit. However, Hudson et al. (1972) described the tendon of insertion as splitting into two branches, with the anterior branch inserting on the anterodorsal edge of the second phalanx of the major digit to the base of the third phalanx of the major digit, and the posterior branch inserting onto the base of the second phalanx of the major digit (Figure 9; Table S1).

#### 3.2.44 M. interosseus ventralis

The counterpart to M. interosseus dorsalis, this muscle's origin is on the caudoventral surface of the major metacarpus and the cranioventral margin of the minor metacarpus. It fills the ventral part of the intermetacarpal space and is craniocaudally wider than M. interosseus dorsalis. We were unable to find the tendons of insertion. Hudson et al. (1972) reported that it inserts mainly near the distal end of the distal phalanx of the major digit in their specimens, whereas Suzuki et al. (2014) reported that it inserts on the proximal portions of the cranial surfaces of the proximal and distal phalanges of the major digit in *Eudromia elegans* (Figure 9; Table S1).

#### 3.2.45 M. flexor digiti minoris

This tiny muscle's origin is on the caudal margin of the minor metacarpus. It extends to the caudal edge of the phalanx of the minor digit, where it becomes tendinous at its insertion (Figure 9; Table S1).

## 4 DISCUSSION

### 4.1 Pectoral flight musculature

Similar to many galliforms, tinamids have relatively small wingspans compared to their body size, and high wing loadings (a measure of body mass supported by unit wing area; Rayner, 1988). This combination is often seen in predominantly ground‐dwelling birds: reduced wingspans may facilitate flight in densely vegetated habitats, while high loading is associated with rapid flight, which may be important to escape terrestrial predators (Rayner, 1988). Despite the utility of this combination for ground‐dwelling birds, this combination renders flight energetically costly (Rayner, 1988). As a result, tinamous frequently flee threatening situations on foot, and may only fly as a last resort (Forshaw, 1991). Among volant birds, tinamous exhibit a notably high ratio of M. pectoralis mass to body mass (0.223), which is associated with rapid takeoff and vertical flight (Rayner, 1988). This ratio is also high in grouse (0.202) and bustards (0.209), while grebes (0.091) and rails (0.097) represent the low end of this range among flying birds (Rayner, 1988). The M. supracoracoideus, which is primarily responsible for powering the upstroke during flight, is also relatively large in relation to body mass in Tinamidae (0.065), which helps to overcome inertia and allows for rapid wingbeats during takeoff (Rayner, 1988). For comparison, this metric is similarly high in grouse (0.058) and pheasants (0.063) (Rayner, 1988). Deeming (2023) noted that tinamous and other burst flying birds have low ratios of pectoralis relative to supracoracoideus mass, a result of the supracoracoideus being larger than in most birds. These specialisations are clearly evident in the *Nothoprocta pentlandii* specimen examined here. Both muscles have broad origins on the caudally elongated sternal carina (Figure 3; Figure S9).

The origins and insertions of these major components of the pectoral flight musculature are the same as in virtually all other extant volant birds (e.g., Bribiesca‐Contreras & Sellers, 2017; Dial, 1992, Dial et al., 1991; George & Berger, 1966; McGowan, 1986; Raikow, 1977, 1978, 1985a, 1985b; Razmadze et al., 2018; Sullivan et al., 2019; Watanabe et al., 2021; Yasuda, 2004), congruent with the dominant perspective that flight in tinamous is homologous with that in extant Neognathae, and was present in the last common ancestor of extant birds. In all flightless palaeognaths with the exception of rheas, the M. supracoracoideus originates on the coracoidal body alone, as the sternal carina has been lost (Jasinoski et al., 2006; Lo Coco et al., 2022; Maxwell & Larsson, 2007; McGowan, 1982). In the greater rhea *Rhea americana*, the origin of this muscle covers a small area of the sternum's cranioventral surface in addition to the coracoid (Lo Coco et al., 2022). The M. pectoralis originates on the lateral margin of the coracoid in the emu *Dromaius novaehollandiae* (Maxwell & Larsson, 2007), the craniolateral surface of the coracoid in the greater rhea (Lo Coco et al., 2022), on both the lateral margin of the coracoid and on the muscular (ventral) surface of the sternal body in the North Island brown kiwi *Apteryx mantelli* (McGowan, 1982), and on the ligamentum sternocoracoideum laterale as well as on the muscular surface of the sternal body in ostriches (Jasinoski et al., 2006). These unusual arrangements contrast with the very large origin of the M. pectoralis on the sternal carina of *N. pentlandii*, which does not contact the coracoid whatsoever (Figure 3; Figure S9).

### 4.2 Comparison of *Nothoprocta pentlandii* and *Eudromia elegans*

Tinamidae is divided into two extant constituent subclades, one generally specialized for life in forested environments (Tinaminae), and the other for more open habitats (Nothurinae; Almeida et al., 2021; Bertelli et al., 2014; Miranda‐Ribeiro, 1938). Species within Nothurinae are generally considered to be stronger fliers than Tinaminae of similar size, likely due to the former's preference for more open habitats with less shelter from potential predators (Bertelli et al., 2014; Fjeldså & Krabbe, 1990). *Nothoprocta pentlandii* (the focal taxon of this investigation), and *Eudromia elegans* (the subject of a recent myological study by Suzuki et al., 2014) both belong to the tinamid subclade Nothurinae (Almeida et al., 2021; Bertelli, 2017; Bertelli et al., 2014). These taxa are estimated to have diverged roughly 20 to 25 million years ago (Almeida et al., 2021; Prum et al., 2015), and exhibit qualitatively similar pectoral musculoskeletal anatomy overall. Several tendons of insertion were not visible in our contrast‐stained specimen due to iodine's lower affinity for tendon as compared with myofiber (Bribiesca‐Contreras & Sellers, 2017). However, the general uniformity of tinamou postcranial morphology (Hudson et al., 1972) facilitated justified inferences about aspects of tendon insertion that were not visible through comparison with data from other tinamou taxa such as *Eudromia*.

There are, however, several examples of interspecific musculoskeletal variation within Nothurinae that are worthy of note as they may have minor effects on flight capacity. The craniolateral processes of the sternum are elongated in the clade composed of *Nothoprocta*, *Taoniscus*, *Nothura*, and *Rhynchotus* as compared with *Eudromia* (Bertelli et al., 2014). An enlarged craniolateral process of the sternum and lateral process of the coracoid have also been suggested to play a role in increased flight capacity in those same taxa with respect to *Eudromia* (Bertelli et al., 2014). In *Nothoprocta pentlandii*, as in other tinamids (Hudson et al., 1972; Suzuki et al., 2014), the ventral surface of the craniolateral process of the sternum hosts the caudal portion of the origin of M. coracobrachialis caudalis, and the dorsal surface hosts the origin of M. sternocoracoideus (Figure 1). The origin of M. coracobrachialis caudalis extends to the lateral process of the coracoid. M. sternocoracoideus functions to retract the coracoid at the coracosternal joint, and M. coracobrachialis caudalis both depresses and rotates the humerus (Raikow, 1985a). Both of these muscles belong to the group of major downstroke muscles in the European starling *Sturnus vulgaris* (Dial et al., 1991). It is possible, therefore, that the increased area of origin for these muscles contributes to a more powerful flight stroke in this subclade of Nothurinae, though this hypothesis will need to await specific biomechanical validation.

### 4.3 Implications for palaeognath and neornithine ancestral states

Although the myology and osteology of *Nothoprocta pentlandii* are broadly similar to those of many burst‐flying neognaths, tinamous differ from neognaths in several features of the flight apparatus. Considering the phylogenetic position of tinamous within Palaeognathae, and the sister group relationship between Palaeognathae and all other crown group birds, differences in the flight apparatus of our specimen may be relevant for reconstructing the ancestral flight apparatus of all crown group birds. Nearly all muscles reported in the flight apparatus of neognaths are present. The only muscle of the flight apparatus of neognaths that is apparently lacking in *N. pentlandii* (as it is in other tinamids), is M. biceps brachii pars propatagialis (the biceps slip in Hudson et al., 1972). Presumably, the full suite of muscles found in the flight apparatus of *N. pentlandii* was present in the last common ancestor of crown group palaeognaths, before many of these muscles were lost or modified among the various ratite lineages (Figure S11a).

The M. entepicondylo‐ulnaris, absent in all Neoaves (Beddard, 1898; Livezey & Zusi, 2006, 2007; Vanden Berge & Zweers, 1993), is present in *N. pentlandii* and all other tinamids studied (Hudson et al., 1972; Suzuki et al., 2014) (Figures 6 and 7). Its presence in non‐tinamid palaeognaths is variable: for example, it is occasionally present in emus, where it is fused along most of its length to the belly of M. flexor carpi ulnaris when it does appear (Maxwell & Larsson, 2007). McGowan (1982) failed to locate this muscle in the North Island brown kiwi, though it was reported by Beddard (1898), suggesting that its presence in kiwi may also be polymorphic. It was not found in the greater rhea by Lo Coco et al. (2022). Because this muscle is only known to be present in some Palaeognathae and Galloanserae, we can surmise that it was lost along the stem lineage of Neoaves (as per Livezey & Zusi, 2006, 2007; Figure S11).

In neognaths, M. subcoracoideus [which depresses and rotates the humerus dorsally (Raikow, 1985a)] is subdivided into the caput dorsale and caput ventrale, though both heads are not present in all species (George & Berger, 1966; Jasinoski et al., 2006; McGowan, 1986). The caput dorsale's origin is on the medial side of the coracoid, the omal end of the furcula, the acromion of the scapula, and/or adjacent ligaments, while the caput ventrale originates on the dorsocaudal surface of the coracoid, the cranial margin of the sternum, and/or adjacent ligaments (George & Berger, 1966; Raikow, 1977, 1985b; Razmadze et al., 2018). Only one head has ever been observed in palaeognaths (Hudson et al., 1972; Jasinoski et al., 2006; Lo Coco et al., 2022; Maxwell & Larsson, 2007; McGowan, 1982), and we confirm the presence of only one head of the M. subcoracoideus in *N. pentlandii* (Figures 1 and 4). This originates on the caudolateral margin of the coracoid in the emu (Maxwell & Larsson, 2007), the caudomedial edge of the coracoid in ostriches (Jasinoski et al., 2006), the craniolateral process of the sternum and lateral and distal edge of the coracoid in the greater rhea (Lo Coco et al., 2022), and the medial surface of the coracoid in the North Island brown kiwi, when present [the presence of this muscle is individually and contralaterally variable in this species (McGowan, 1982)]. Interestingly, despite contacting the coracoid, the M. subcoracoideus has no apparent origin on that bone in *N. pentlandii*, nor does it in any other tinamid examined (Hudson et al., 1972; Suzuki et al., 2014; Figures 1 and 4). Its origin on the internal rostral spine of the sternum in tinamids therefore appears unique among extant palaeognaths. With the information available at present, it is impossible to conclude with confidence whether this muscle's sternal origin represents a tinamid synapomorphy, or retention of the plesiomorphic condition for Neornithes (Figure S11a).

As in other tinamids (Hudson et al., 1972; Suzuki et al., 2014), we observed two heads of M. subscapularis, the caput mediale and caput laterale (Figures 1 and 4). Only the caput mediale of M. subscapularis is present in cassowaries, North Island brown kiwi, ostriches, and emu (Beddard, 1898; Jasinoski et al., 2006; Maxwell & Larsson, 2007; McGowan, 1982), while in the greater rhea, the caput laterale instead appears to be present (Lo Coco et al., 2022). Both heads are present in Neognathae (Dial et al., 1991; Jasinoski et al., 2006; Raikow, 1977; Razmadze et al., 2018; Yasuda, 2004). With this information, it appears likely that two heads for this muscle represents the ancestral condition for Neornithes, with multiple independent reductions to a single head having taken place among the flightless palaeognaths (Figure 11a).

In most neognaths, the insertion of the M. pronator profundus is said to extend as far or further distally than that of the M. pronator superficialis (e.g., George & Berger, 1966; McGowan, 1986; Raikow, 1977; Razmadze et al., 2018; Watanabe et al., 2021), and the former muscle is further subdivided into two bellies in some passerines (Raikow, 1978, 1985b). However, the opposite is true in *N. pentlandii* (Figures 6 and 7), and Hudson et al. (1972) also made note of the great length of the M. pronator superficialis in several tinamid genera. Most other palaeognaths only have one pronator (Beddard, 1898; Maxwell & Larsson, 2007; McGowan, 1982). Although only variably present in the emu (Maxwell & Larsson, 2007), when present, the authors assigned the lone pronator to M. pronator superficialis, and the single pronator in the North Island brown kiwi was also identified as the M. pronator superficialis (McGowan, 1982). Lo Coco et al. (2022) reported both pronators in the greater rhea but state that they share origin and insertion, cannot be separated, and are largely undifferentiated. Based on the presence of two pronators in tinamous, neognaths, and possibly the greater rhea, we infer that the ancestral neornithine was similarly characterised by two pronators, but, as a result of uncertainty in character optimization, we cannot comment on the relative distal extent of the two pronators in the last common ancestor of Neornithes (Figure S11a).

The M. scapulohumeralis cranialis is not present in kiwi and ostriches (Jasinoski et al., 2006; McGowan, 1982), but is present in the greater rhea and variably present in the emu in which it is sometimes fused with M. scapulohumeralis caudalis (Lo Coco et al., 2022; Maxwell & Larsson, 2007). It is present in a phylogenetically widespread sample of neognaths (e.g., *Cygnus*, *Gallus*, *Gallirallus*, *Alca*, *Sturnus*, *Loxops*), but not others (e.g., *Buteo*, *Pelecanus*, *Spheniscus*, *Psittacus*; Beddard, 1898; Dial et al., 1991; Jasinoski et al., 2006; McGowan, 1986; Raikow, 1977; Razmadze et al., 2018; Vanden Berge & Zweers, 1993; Watanabe et al., 2021; Yasuda, 2004). It was observed in all 31 galliform genera examined by Hudson and Lanzillotti (1964). When present, it originates on the lateral aspect of the scapular neck and inserts between the two heads of the M. humerotriceps distal to the dorsal leg of the pneumotricipital fossa (Jasinoski et al., 2006; Vanden Berge & Zweers, 1993). The greater rhea appears to represent an exception, as Lo Coco et al. (2022) report its origin as “the inner edge of the scapular blade on its medial surface”. The former is precisely the condition we observed in *N. pentlandii* (Figures 1, 2 and 4), and therefore we consider this condition to be most likely representative of the ancestral state for Neornithes. The presence of the M. latissimus dorsi pars metapatagialis (Figure S8) follows a similar phylogenetic pattern, though its presence is generally uncommon (Vanden Berge & Zweers, 1993). It is absent in some but not all neognaths (McGowan, 1986; Raikow, 1977; Razmadze et al., 2018) and palaeognaths (Beddard, 1898). Among Galliformes, it was absent in Cracidae, but present in all other genera examined (Hudson & Lanzillotti, 1964). Based on its presence in tinamous, galliforms, and some neoavians (e.g., *Gallirallus*, *Gavia*, *Larus*; McGowan, 1986; Watanabe et al., 2021), we likewise conclude that this muscle was present in the most recent common ancestor of crown birds.

Our digital dissection revealed an extremely well‐developed M. coracobrachialis cranialis in *N. pentlandii* (Figures 1, 2 and 5), which is consistent with reports from other tinamids (Hudson et al., 1972; Suzuki et al., 2014). McGowan (1982) noted that this muscle is small in kiwi, but large in ostriches, and it is also large in the greater rhea (Lo Coco et al., 2022). In tinamids, the muscle can be divided into a short portion that inserts just proximal to the insertion of the M. pectoralis, and a long portion that extends distally over much of the cranial humeral shaft. Typically, its insertion is confined to a fossa on the intertubercular plane of the humerus in neognaths (Vanden Berge & Zweers, 1993), which is the case in *Gallus* (Yasuda, 2004) and in all galliforms examined by Hudson and Lanzillotti (1964). The absence of this expanded insertion in Galliformes, which contains many burst‐flying taxa, suggests that it does not represent a necessary specialization for burst flight. It is entirely absent or vestigial in some passerines (Raikow, 1977, 1978, 1985b). Again, we cannot conclude with confidence whether this expanded insertion covering the cranial humeral shaft is a tinamid synapomorphy, or if it is indicative of the ancestral state of Neornithes.

The M. interosseous dorsalis appears to insert on the minor digit in *N. pentlandii* (Figure 9) as well as those observed by Suzuki et al. (2014) instead of the major digit as found by Hudson et al. (1972), though we note that the distal portion of the tendon of insertion cannot be visualized at the point of attachment. An insertion on the major digit for this muscle has been observed in a diverse sample of neognaths. The insertion occurs on the proximal margin of the craniodorsal surface of the second phalanx of the major digit in *Gallus* (Yasuda, 2004), the Hawaiian honeycreeper *Loxops virens* (Raikow, 1977), and the parrot *Psittacus erithacus* (Razmadze et al., 2018), whereas it occurs on the proximal margin of the craniodorsal surface of the second phalanx of the major digit in the flightless rail *Gallirallus australis* (McGowan, 1986) and the Eurasian woodcock *Scolopax rusticola* (Watanabe et al., 2021), and the dorsal apex of the proximal margin of the second phalanx of the major digit in the razorbill *Alca torda* (Watanabe et al., 2021). Within Palaeognathae, the only non‐tinimid in which it appears is the greater rhea, in which its tendon splits and inserts on both the first and second phalanxes of the major digit (Lo Coco et al., 2022). This muscle is absent in all other non‐tinamid palaeognaths (McGowan, 1982), but its presence in the greater rhea, tinamous, and neognaths suggests that it was present in the last common ancestor of crown birds. We suggest that future work should clarify the point of insertion for this muscle in tinamous, as an insertion on the minor digit has not been reported in any other bird taxon and thus could represent a myological synapomorphy of Tinamidae. If our interpretation, and the observation of Suzuki et al. (2014) is indeed accurate, it would not be possible to reliably infer whether this muscle inserted on the minor digit or major digit in the last common ancestor of Neornithes.

### 4.4 Flight and physiology in stem palaeognaths and ancestral crown birds

In adult ratites, the scapula and coracoid are co‐ossified as a single element [the scapulocoracoid; (Jasinoski et al., 2006; Lo Coco et al., 2022; Maxwell & Larsson, 2007; McGowan, 1982)]. In *Nothoprocta pentlandii*, as in other tinamids, there is no evidence of fusion between these two bones (Hudson et al., 1972; Suzuki et al., 2014; Figures S1 and S2). In the seemingly implausible scenario in which all palaeognaths were descended from a ratite‐like common ancestor and tinamous secondarily regained flight, numerous anatomical reversals would need to have arisen along the tinamid stem lineage in order to give rise to the flight‐capable tinamid skeleton, including the secondary decoupling of the scapulocoracoids, re‐acquisition of a sternal carina, and shifting of the major flight muscle origins back to the sternal carina from the coracoid and the sternal body. While we consider such a scenario highly unlikely, we explore this hypothetical scenario in more detail below.

With tinamous largely accepted to be phylogenetically nested within the traditional ‘Ratitae’, and assuming a flying last common ancestor for Neornithes, the most parsimonious explanation for the distribution of powered flight among palaeognaths would be a single loss of flight among stem palaeognaths and a reacquisition of flight among stem tinamous. Indeed, a reacquisition of flight along the ancestral lineage subtending the tinamou‐moa clade was favoured in maximum likelihood analyses investigating this question (Phillips et al., 2009). We obtained similar results from our own maximum parsimony analysis, with a reacquisition of traits associated with flight, such as a sternal keel and a decoupled scapulocoracoid, arising along the tinamou stem lineage (Figure S11b). Dollo's Law is often invoked to argue against a secondary origin of neornithine flight among palaeognaths, and indeed there are no known examples of secondary flight acquisition among crown birds (Harshman et al., 2008; Smith et al., 2012). However, a growing number of exemptions to the expectations of Dollo's Law have become apparent (Collin & Miglietta, 2008), including the apparent re‐acquisition of digits in *Bachia* lizards (Kohlsdorf & Wagner, 2006), wings in stick insects (Whiting et al., 2003), and coiled shells in slipper limpets (Collin & Cipriani, 2003). Despite the multiple lines of biogeographic and myological evidence previously discussed, rejection of the parsimonious hypothesis that flightlessness evolved once in ratites and was reversed in tinamous requires evidence for independent acquisition of morphological traits related to flightlessness in ratites (Suzuki et al., 2014). Such evidence, though sparse, has begun to accrue in recent years. For example, developmental trajectories of the wings of ostrich and emu embryos suggest that alternative heterochronic pathways may have given rise to flightlessness in these distantly related palaeognath lineages (Faux & Field, 2017). Furthermore, cardiac transcription factor *Nkx2.5* is expressed in the developing wings of emu embryos but not in those of ostriches; mis‐expression of this transcription factor in limb buds is associated with reduced wing growth in embryonic chickens (Farlie et al., 2017). Given that convergent morphological responses related to flightlessness have been well documented in other neornithine clades, particularly rails (Gaspar et al., 2020), it stands to reason that the so‐called ‘ratite’ condition may well have emerged independently multiple times from volant ancestors.

Given the strong body of evidence from phylogenetic (Baker et al., 2014; Chojnowski et al., 2008; Claramunt & Cracraft, 2015; Grealy et al., 2017; Hackett et al., 2008; Harshman et al., 2008; Kuhl et al., 2020; Mitchell et al., 2014; Phillips et al., 2009; Prum et al., 2015; Smith et al., 2012; Yonezawa et al., 2017), developmental (Bickley & Logan, 2014; Farlie et al., 2017; Faux & Field, 2017), and genomic and mitogenomic studies (Cloutier et al., 2019; Feng et al., 2020; Kimball et al., 2019; Reddy et al., 2017; Sackton et al., 2019; Takezaki, 2023; Urantówka et al., 2020; Wang et al., 2021) that ratites are paraphyletic with respect to tinamous, and the expectation that the last common ancestor of palaeognaths was volant, the present dataset helps provide a starting point to begin reconstructing attributes of the flight apparatus of the hypothetical volant last common ancestor of crown Palaeognathae. Molecular divergence time studies (Almeida et al., 2021; Claramunt & Cracraft, 2015; Grealy et al., 2017; Jarvis et al., 2014; Yonezawa et al., 2017) and direct fossil evidence (Clarke et al., 2005; Field et al., 2020) illustrate that the ancestral divergence between palaeognaths and neognaths occurred prior to the K–Pg mass extinction, presumably at some point in the Late Cretaceous. Unfortunately, unambiguous evidence of stem group palaeognaths from the Cretaceous and earliest Palaeocene is presently unknown (Hope, 2002; Mayr, 2009; Parris & Hope, 2002). Lithornithidae, a group of late Palaeocene and early to mid‐Eocene fossil total‐group palaeognaths (Houde, 1988; Nesbitt & Clarke, 2016; Widrig & Field, 2022; Yonezawa et al., 2017) may represent the best group upon which to base such reconstructions, as they represent the only presumably volant palaeognaths known outside of Tinamidae (Houde, 1988; Nesbitt & Clarke, 2016; Torres et al., 2020; Widrig & Field, 2022). Lithornithids are most often recovered as the sister clade of tinamous in morphology‐only phylogenetic analyses due to numerous skeletal similarities that tend to optimize as synapomorphies of a Tinamidae + Lithornithidae clade (Nesbitt & Clarke, 2016). The body size distributions of lithornithids and tinamids are comparable, and the skulls of some lithornithids and tinamids bear some striking similarities, hinting at some possible similarities in foraging behaviour (Houde, 1988). However, the results of morphology‐only phylogenetic analyses are prone to distortion by anatomical homoplasy (e.g., Chen et al., 2019; Gaspar et al., 2020; Goloboff et al., 2008; Mayr, 2008; Steell et al., 2022; Wake, 1991; Yonezawa et al., 2017). As evinced by the multiple apparently independent origins of large size and flightlessness in palaeognaths (Mitchell et al., 2014; Yonezawa et al., 2017), this clade would appear to be especially susceptible to topological inaccuracies induced by convergence. Indeed, when the same anatomical datasets are reanalysed under molecular topological constraints, the inferred position of Lithornithidae shifts to the palaeognath stem lineage, where they are inferred to represent the monophyletic sister taxon of other palaeognaths (Nesbitt & Clarke, 2016). Maximum‐likelihood trees inferred using characters exhibiting low homoplasy also support a position on the palaeognath stem for Lithornithidae (Yonezawa et al., 2017).

All known lithornithid fossils have been found in North America and Europe, contrasting with the predominantly Gondwanan distribution of extant palaeognaths (Houde, 1988; Nesbitt & Clarke, 2016; Stidham et al., 2014). A scapula possibly belonging to a lithornithid was found near the K–Pg boundary in New Jersey, but further information is needed to confirm its phylogenetic affinities as well as its precise age (Hope, 2002; Mayr, 2009; Parris & Hope, 2002). The oldest‐known unambiguous lithornithid fossils are from the middle Palaeocene of North America, suggesting a possible Northern Hemisphere origin for palaeognaths (Houde, 1986, 1988; Yonezawa et al., 2017). Regardless of their exact geographic origin, palaeognaths appear to be one of many extant avian clades whose extant representatives exhibit very different geographic distributions to those of their early stem group representatives (e.g., Field & Hsiang, 2018, Ksepka et al., 2017, Ksepka & Clarke, 2009, Mayr, 2004, Mayr et al., 2011, Olson, 1987, Saupe et al., 2019).

Despite numerous osteological similarities in the cranium, scapula, and manus (Nesbitt & Clarke, 2016), multiple lines of evidence suggest that lithornithids may have differed in their flight style from extant tinamids. This is of potential biogeographic significance: a capacity for long‐distance flight among early palaeognaths may be necessary to explain the phylogenetic interrelationships of extant palaeognaths (Almeida et al., 2021; Baker et al., 2014; Chojnowski et al., 2008; Claramunt & Cracraft, 2015; Cloutier et al., 2019; Feng et al., 2020; Grealy et al., 2017; Hackett et al., 2008; Harshman et al., 2008; Kimball et al., 2019; Kuhl et al., 2020; Phillips et al., 2009; Prum et al., 2015; Reddy et al., 2017; Sackton et al., 2019; Smith et al., 2012; Takezaki, 2023; Urantówka et al., 2020; Wang et al., 2021; Yonezawa et al., 2017) in light of their presumably Cenozoic divergence times (Almeida et al., 2021; Berv & Field, 2018; Grealy et al., 2017; Prum et al., 2015; Yonezawa et al., 2017) and present‐day biogeographic patterns (Hosner et al., 2017). Long‐distance dispersal among stem palaeognaths would presumably have been unlikely if such taxa exhibited flight capabilities similar to those of extant tinamous [i.e., specialisation for short‐distance burst flight; (Hosner et al., 2017)]. Lithornithids exhibit a relatively short sternum (Houde, 1988), unlike the caudally elongated sternum seen in tinamids (Figure S4), which provides ample attachment area for the extremely large M. pectoralis and M. supracoracoideus underlying their penchant for burst flight (Houde, 1988). Notable differences are also present in the humeri of these two clades. In tinamids, a relatively short humerus and a proximally‐positioned insertion of M. pectoralis (Figures 1, 2, 3 and 5; Figure S9) aids in the rapid wingbeats needed for quick takeoff. The humerus of lithornithids is comparatively long, with a more distally positioned insertion area for M. pectoralis on a prominent deltopectoral crest (Houde, 1988). Together, these osteological traits are indicative of a mode of flight involving slower wingbeats in lithornithids than in tinamous (Houde, 1988; Rayner, 1988).

An exceptionally well‐preserved specimen of the early Eocene lithornithid *Calciavis grandei* from the Green River Formation supports the hypothesis of differing flight styles in tinamids and lithornithids (Torres et al., 2020). Carbonized feather traces enabled an estimate of wing surface area, and body mass was estimated based on the length of the humerus following published scaling equations (Field et al., 2013). The estimated wing loading of *C. grandei* was half that of the red‐winged tinamou *Rhynchotus rufescens*, indicating that far less power was required to take off and sustain flight in *C. grandei* (Torres et al., 2020). The aspect ratio of the reconstructed wing of *C. grandei* was also higher than that of *R. rufescens*, indicating a greater capacity for lift generation. The authors concluded that *C. grandei* may have been capable of sustained flapping flight over long distances, and may have been seasonally migratory (Torres et al., 2020).

Intriguingly, tinamous exhibit the smallest hearts relative to body size of any living birds (Bishop, 1997). Heart size in nine species of tinamou averaged 0.30% of body mass, while in twenty‐two species of Phasianidae (Galliformes) this metric averaged 0.81%. The small size of tinamou hearts has been suggested to hamper aerobic performance in comparison to birds of similar body size and flight style (Altimiras et al., 2017). When subjected to a ‘chase‐and‐exhaust’ challenge, tinamous were shown to accumulate significant amounts of lactate and reach exhaustion in a shorter amount of time than chickens (Altimiras et al., 2017). The rate of cardiac growth relative to body mass remains the same from embryonic to adult stage in two species of tinamids (*Nothoprocta perdicaria* and *N. ornata*) and the American alligator *Alligator mississippiensis*. On the other hand, cardiac growth rate relative to body mass increases throughout development in chickens (Altimiras et al., 2017). Cardiac performance and growth rate in tinamous and alligators were hypothesised to reflect those of ancestral archosaurs, perhaps indicating that the small size of tinamou hearts reflects the plesiomorphic archosaurian condition (Altimiras et al., 2017).

These observations raise alternative possibilities related to the evolution of cardiac performance in crown birds. Perhaps the proportionally larger hearts of neognaths reflect an apomorphic transition enabling improved sustained flight in this clade (implying that the last common ancestor of crown birds had a small, tinamou‐like heart). Alternatively, the small hearts of tinamous may reflect an apomorphic shift related to their less aerobically challenging, cryptic mode of life—implying a large, neognath‐like heart in the most recent neornithine common ancestor. These scenarios have important implications for understanding patterns of palaeognath evolution and dispersal. If tinamou‐like small hearts represent the ancestral condition for Neornithes, application of the Extant Phylogenetic Bracket (Witmer, 1995) would imply that lithornithids were unlikely to have been capable of dispersal via long‐distance flight. However, this notion is challenged by the fact that large hearts comparable to those of neognaths are found in flightless palaeognaths such as ostriches, emu, and rheas (Altimiras et al., 2017). If small hearts were plesiomorphic for crown birds, this would imply that larger hearts in ratites arose multiple times as they transitioned to cursorial lifestyles. Moreover, the interpretation of a relatively small heart as a neornithine plesiomorphy is at odds with interpretations of the palaeobiology of crownward Mesozoic stem birds. For example, *Ichthyornis*, a Late Cretaceous ornithurine that is among the closest‐known relatives of crown birds among non‐crown avialans, is widely assumed to have exhibited an ecology similar to that of extant volant seabirds in light of factors such as its essentially modern postcranial anatomy, general resemblance to extant marine birds, and fossils deriving from open marine deposits which imply a capacity for sustained flight (Benito, Chen, et al., 2022; Clarke, 2004; Demuth et al., 2022; Field, Hanson, et al., 2018; Lowi‐Merri et al., 2023; Marsh, 1880). Therefore, on balance, the hypothesis that the last common ancestor of Neornithes had a relatively large heart, with reduced cardiac capacity representing a tinamou synapomorphy, would seem to be most congruent with multiple lines of evidence.

## 5 CONCLUSIONS

The dataset presented here will help provide a useful reference point for reconstructing the flight apparatus of early palaeognaths, and, by providing a comparison to the well‐characterised morphology of the neognath flight apparatus (e.g., Bribiesca‐Contreras & Sellers, 2017; Dial, 1992; Dial et al., 1991; George & Berger, 1966; McGowan, 1986; Raikow, 1977, 1978, 1985a, 1985b; Razmadze et al., 2018; Sullivan et al., 2019; Watanabe et al., 2021; Yasuda, 2004), help ground reconstructions of the flight apparatus of Mesozoic stem birds and early neornithines. Future reconstructions of the lithornithid flight apparatus will help clarify whether these birds were indeed capable of long‐distance flight, which in turn will help answer questions regarding the ancestral condition of the flight apparatus and physiology of the ancestral crown bird.

Stem palaeognaths (possibly similar in their biology to known lithornithids) appear to be one of a limited number of crown bird lineages to have survived the K–Pg mass extinction event (e.g., Clarke et al., 2005; Field et al., 2020; Field, Bercovici, et al., 2018; Grealy et al., 2017, Prum et al., 2015). Therefore, a more advanced understanding of lithornithid palaeobiology may help elucidate the factors involved in enabling the survivorship of birds across this mass extinction event and their diversification in its aftermath. Given the phylogenetic position of palaeognaths as the sister clade to all other crown birds, such efforts also have important potential to shed light on the nature of the last common ancestor of crown birds.

## AUTHOR CONTRIBUTIONS

All authors conceptualised the project. Bhart‐Anjan S. Bhullar scanned the specimen. Klara E. Widrig performed digital segmentation, made the figures, and wrote the first draft. All authors reviewed and edited the final manuscript. Daniel J. Field supervised the project.

### OPEN RESEARCH BADGES

This article has earned an Open Data badge for making publicly available the digitally‐shareable data necessary to reproduce the reported results. The data is available at https://www.morphosource.org/projects/000529384.

## Supporting information


Data S1.

## Data Availability

The data that support the findings of this study are openly available on Morphosource at https://www.morphosource.org/projects/000529384, project ID number 000529384.
