# Supplementary material for: 3D atlas of tinamou (Neornithes: Tinamidae) pectoral morphology: Implications for reconstructing the ancestral neornithine flight apparatus
Source: J Anat. 2023 Jun 26;243(5):729–57. doi: 10.1111/joa.13919 (PMC10557402; doi:10.1111/joa.13919)
Supplement: Supplementary file 1 — Data S1. [file JOA-243-729-s001.docx]

**Supplementary Information**

**
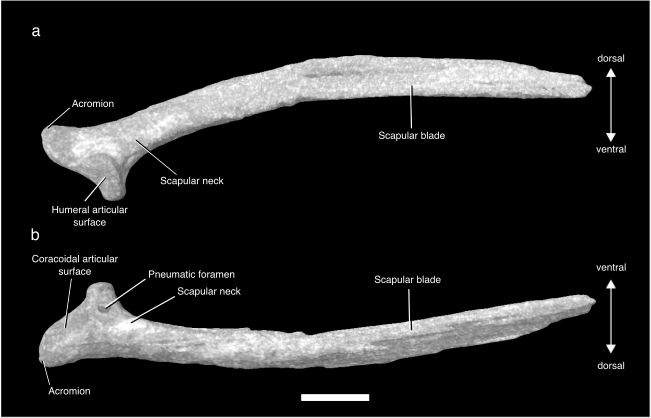
**

**SI Figure 1: Digitally segmented scapula of *Nothoprocta pentlandii*.** A: left scapula, dorsolateral view. B: same, ventromedial view. Scale bar, 5 mm.


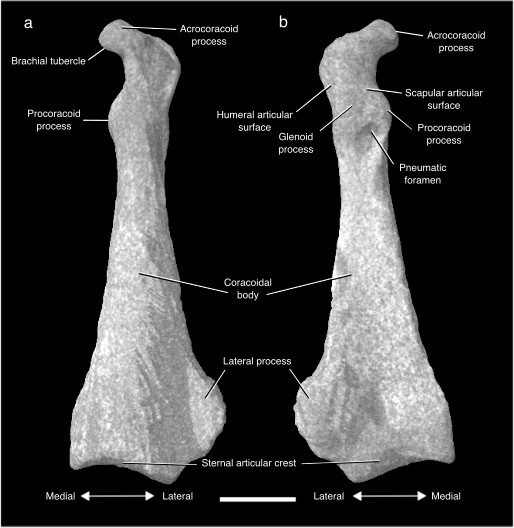


**SI Figure 2: Digitally segmented coracoid of *Nothoprocta pentlandii*.** A: left coracoid, ventral view. B: same, dorsal view. Scale bar, 5 mm.


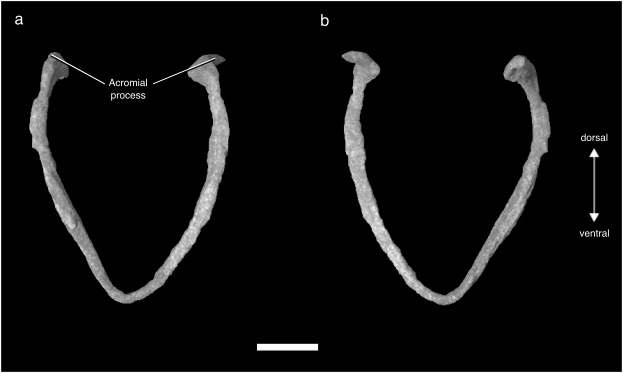


**SI Figure 3: Digitally segmented furcula of *Nothoprocta pentlandii.*** A: furcula, ventrocranial view. B: same, dorsocaudal view. Scale bar, 5 mm.


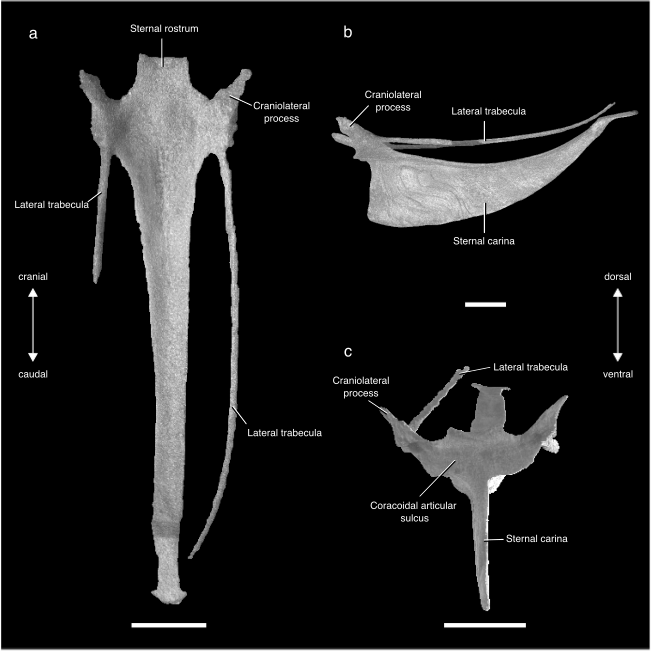


**SI Figure 4: Digitally segmented sternum of *Nothoprocta pentlandii.*** The caudal two-thirds of the left lateral trabecula is not visible in our specimen, perhaps as a result of displacement during formalin injection prior to contrast staining. A: sternum, dorsal view. B: same, left lateral view. C: same, cranial view. Scale bars, 10 mm.


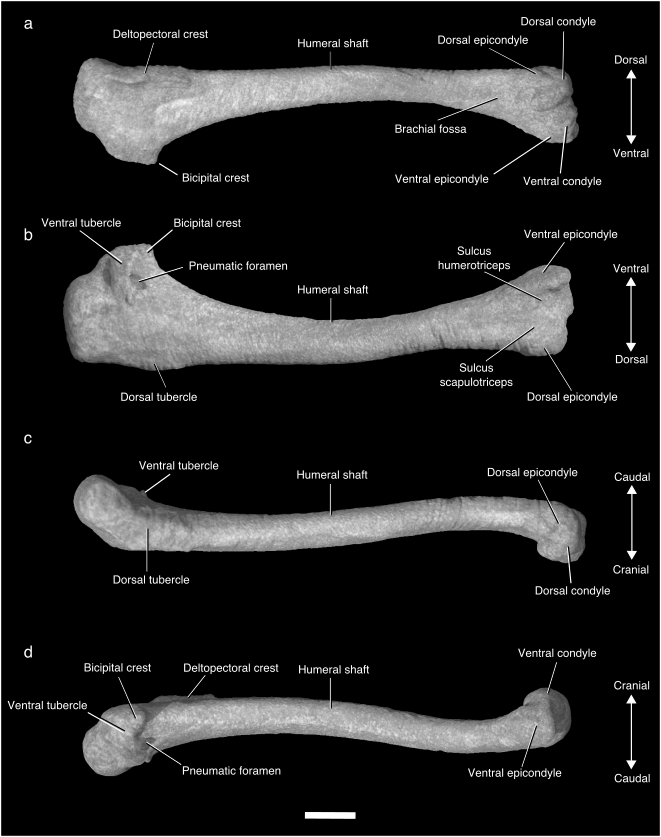


**SI Figure 5: Digitally segmented humerus of *Nothoprocta pentlandii*.** A: left humerus, cranial view. B: same, caudal view. C: same, dorsal view. D: same, ventral view. Scale bar, 5 mm.

**
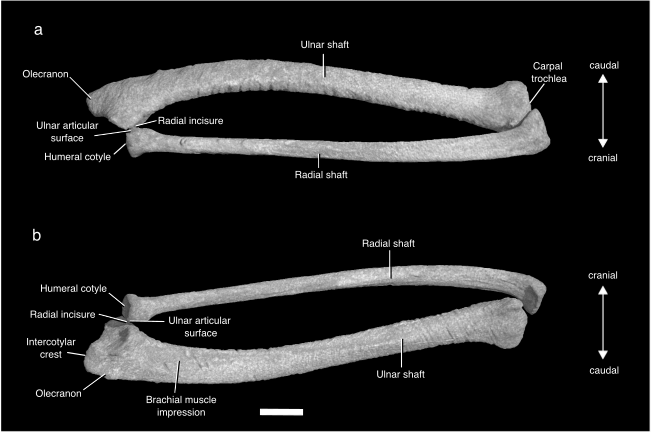
**

**SI Figure 6: Digitally segmented forearm of *Nothoprocta pentlandii.*** A: left radius and ulna in original articulation in a flexed position, dorsal view. B: same, ventral view. Scale bar, 5 mm.


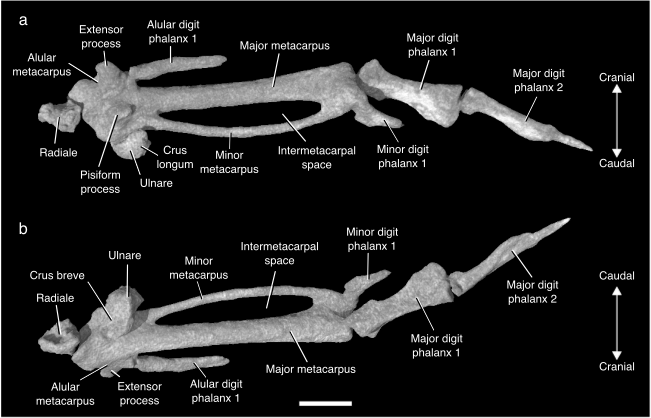


**SI Figure 7: Digitally segmented manus of *Nothoprocta pentlandii.*** A: left radiale, ulnare, carpometacarpus, and phalanges in original articulation, ventral view. B: same, dorsal view. Scale bar, 5 mm.


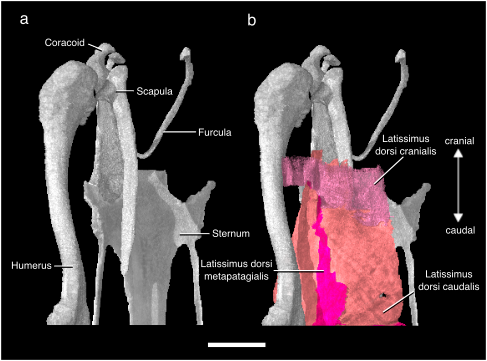


**SI Figure 8: Digitally segmented muscles of the latissimus dorsi complex of *Nothoprocta pentlandii*.** A: left humerus, left coracoid, left scapula, furcula, and sternum. B: same as A, with Mm. latissimus dorsi pars cranialis, pars caudalis, and pars metapatagialis added. All are displayed in original articulation in dorsocaudal view. Scale bar, 10 mm.


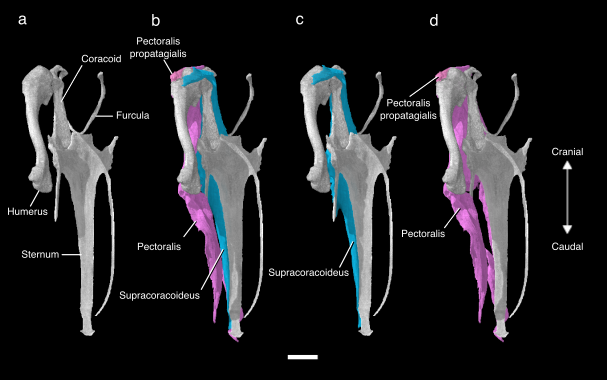


**SI Figure 9: Digitally segmented pectoral flight muscles of *Nothoprocta pentlandii*.** A: sternum, furcula, left coracoid, left humerus. B: same as A, with left Mm. supracoracoideus, pectoralis, and pectoralis pars propatagialis added. C: same as A, with left M. supracoracoideus added. D: same as A, with left Mm. pectoralis and pectoralis pars propatagialis added. All are displayed in original articulation in dorsal view. Scale bar, 10 mm.

**
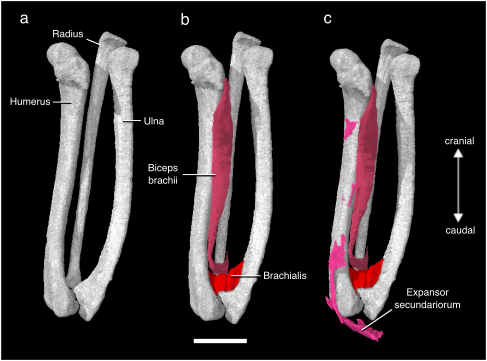
**

**SI Figure 10: Digitally segmented M. biceps brachii and M. brachialis of *Nothoprocta pentlandii*.** A: Left humerus, radius, and ulna. B: same as A, with Mm. biceps brachii and brachialis added. C: same as B, with M. expansor secundariorum added. M. expansor secundariorum is poorly visualized along the humeral shaft in our specimen. All are displayed in original articulation in ventral view. Note that the orientations in this figure refer to those in the flexed wing rather than the standard anatomical orientations for the extended wing. Scale bar, 10 mm.

**
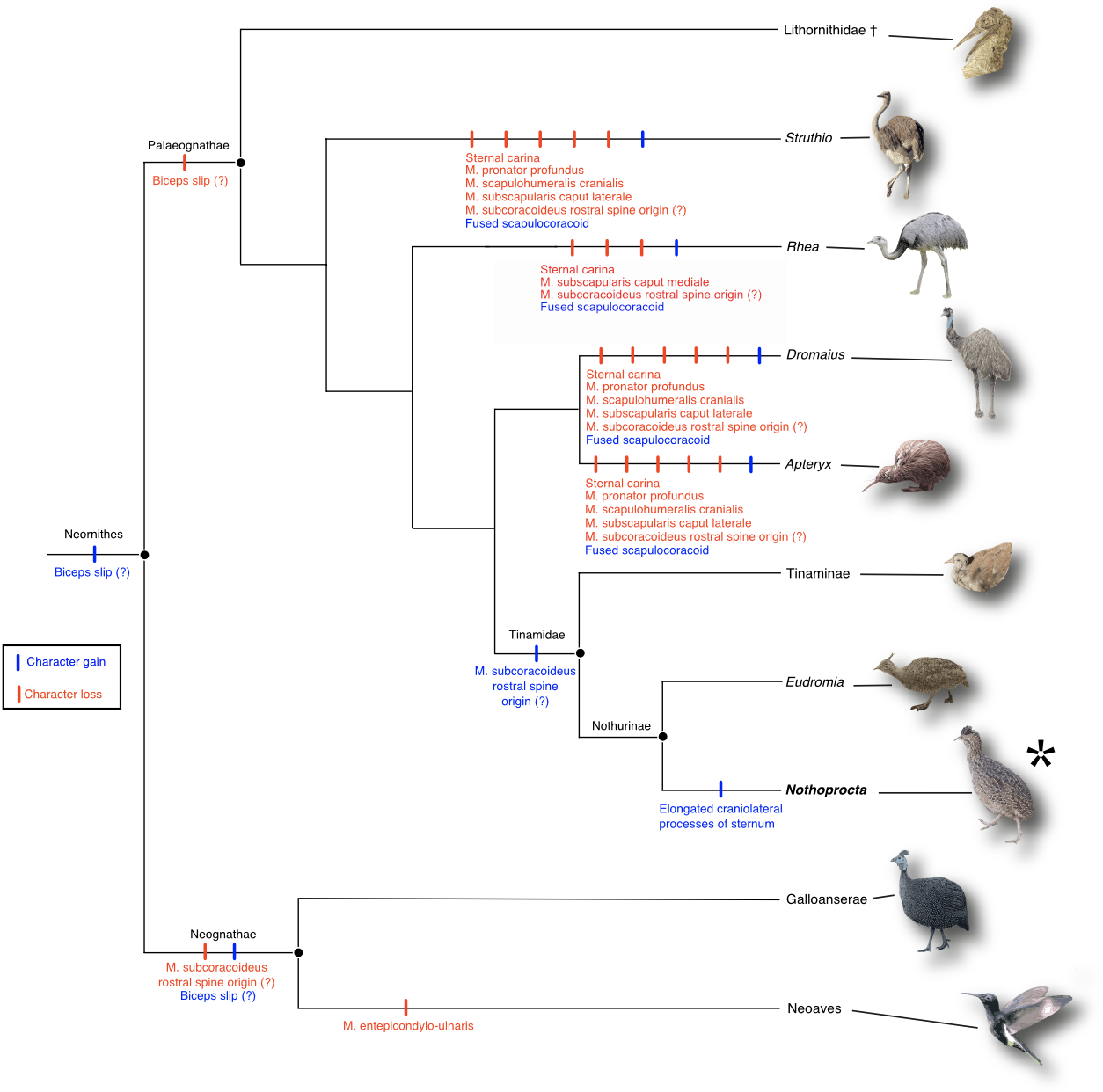
**

**SI Figure 11a: Inferred gains and losses of myological and osteological characters within Neornithes under the assumption of Dollo’s Law.** Tree topology follows that of Mitchell et al. (2014), Prum et al. (2015), and Nesbitt and Clarke (2016). Extinct taxa denoted with daggers. Photos © DJF, except *Eudromia* (© KEW). Holotype skull of *Pseudocrypturus cercanaxius* (USNM336103) is shown for Lithornithidae. Question marks denote characters with ambiguous optimization.

**
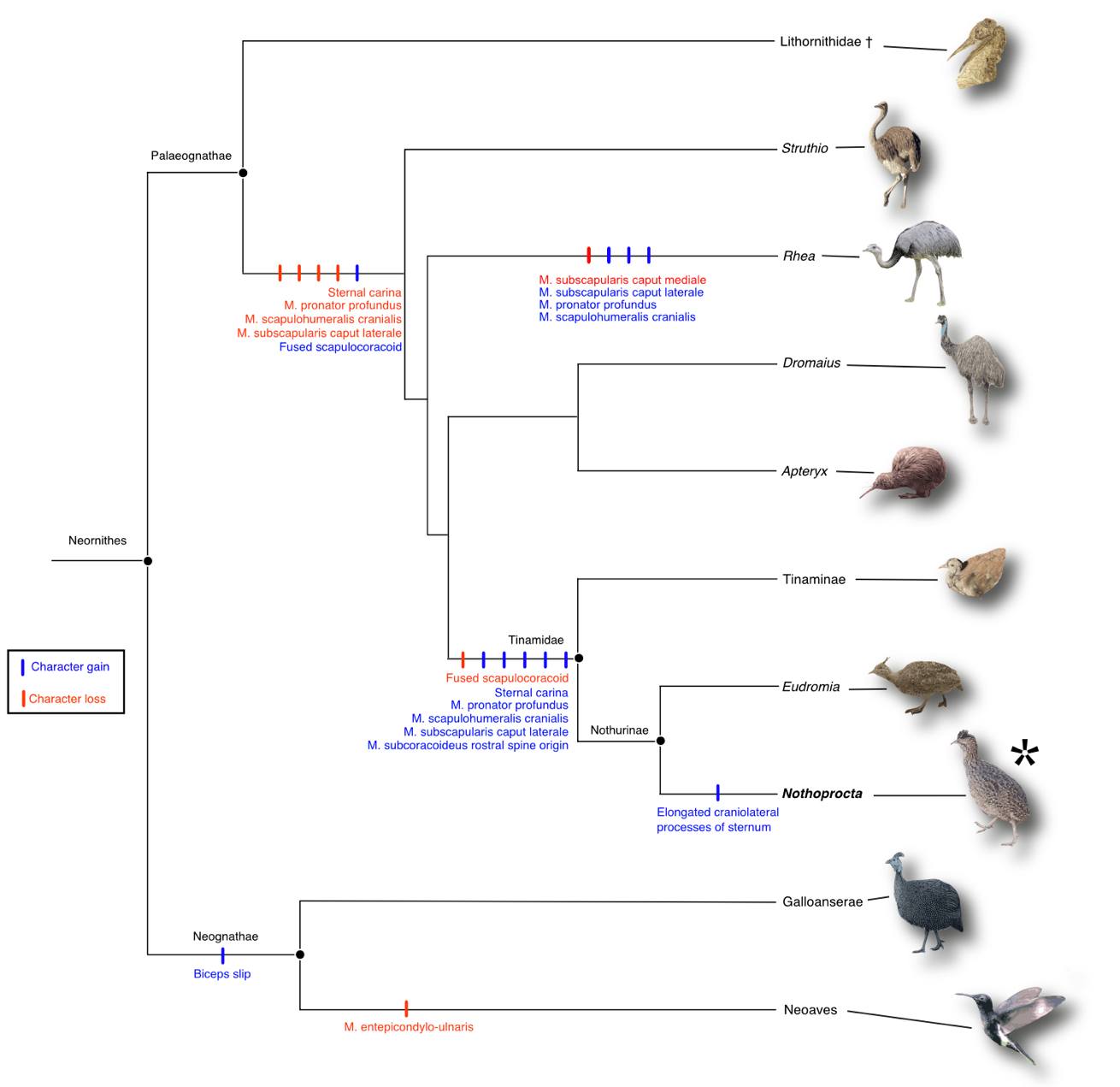
**

**SI Figure 11b: Inferred gains and losses of myological and osteological characters within Neornithes under maximum parsimony.** Maximum parsimony analysis was performed in TNT (Goloboff and Catalano, 2016). Tree topology follows that of Mitchell et al. (2014),Prum et al. (2015), and Nesbitt and Clarke (2016). Extinct taxa denoted with daggers. Photos © DJF, except *Eudromia* (© KEW). Holotype skull of *Pseudocrypturus cercanaxius* (USNM336103) is shown for Lithornithidae.

**
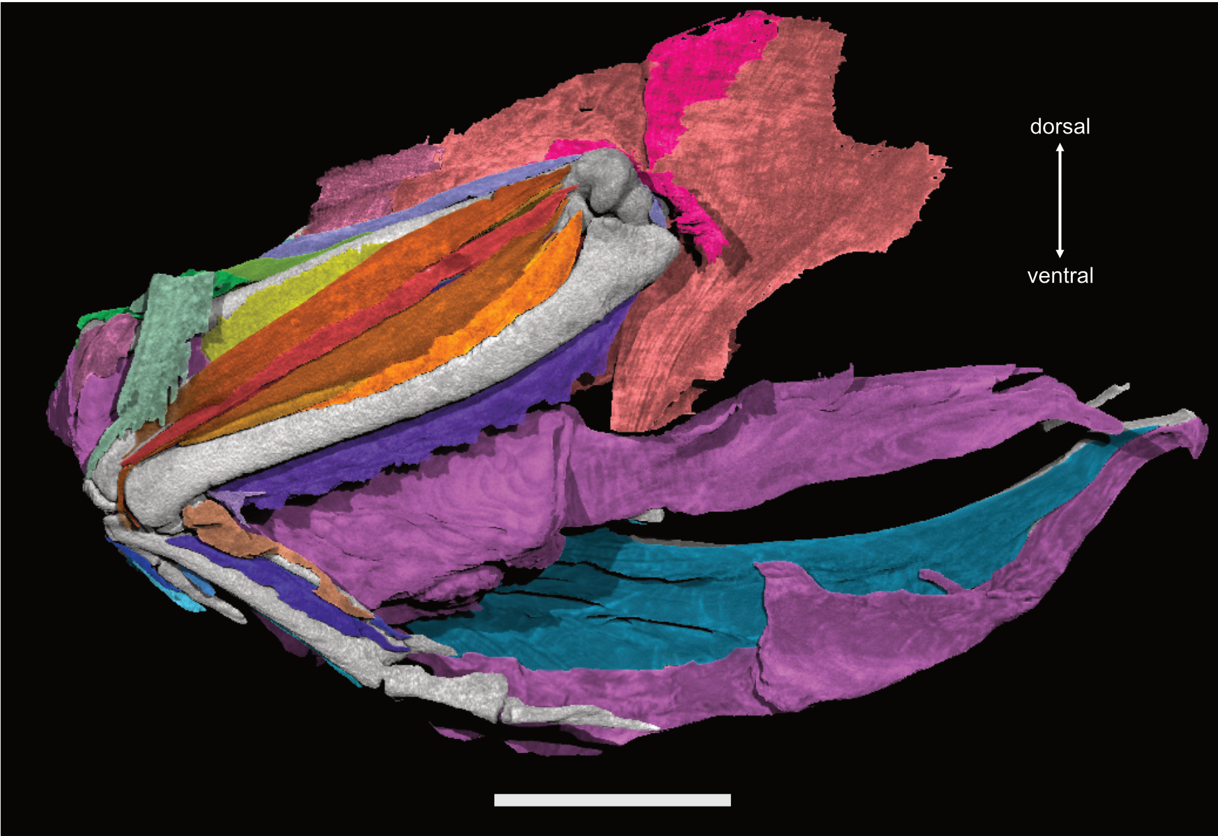
**

**SI Figure 12:** All bones and muscles of the wing apparatus, in left lateral view. Scale bar, 20 mm.

**SI Table 1: Origins and insertions of muscles of the pectoral girdle of *Nothoprocta pentlandii.***

| **Muscle Name** | **Origin** | **Insertion** |
| --- | --- | --- |
| *M. latissimus dorsi pars cranialis* | Spinal processes of thoracic vertebrae II-VI (Suzuki et al., 2014) | Caudal margin of proximal humeral shaft |
| *M. latissimus dorsi pars caudalis* | Spinal processes of caudal thoracic vertebrae, thoracic vertebral ribs II-V, cranial margin of ilium (Hudson et al., 1972) | M. latissimus dorsi pars cranialis, M. scapulotriceps, also M. deltoideus pars major (Hudson et al., 1972) |
| *M. latissimus dorsi pars metapatagialis* | Spinal processes of caudal thoracic vertebrae (Hudson et al., 1972) | Caudal aspect of M. serratus superficialis pars metapatagialis (Hudson et al., 1972) |
| *M. supracoracoideus* | Sternal carina, ventral sternal notch membrane, ventromedial coracoidal body | Dorsal tubercle of humerus |
| *M. pectoralis* | Sternal carina, sternal notch membrane, ventrolateral furcula, lateral trabeculae of sternum | Deltopectoral crest of humerus |
| *M. pectoralis pars propatagialis* | Body of M. pectoralis near deltopectoral crest | Distal M. deltoideus pars propatagialis and cranioventral margin of proximal propatagial ligament |
| *M. sternocoracoideus* | Dorsal and dorsomedial surfaces of craniolateral process of sternum | Dorsal surface of coracoid lateral process |
| *M. scapulohumeralis cranialis* | Lateral surface of scapular neck | Distal edge of pneumatic foramen of humerus |
| *M. scapulohumeralis caudalis* | Lateral scapula | Medial margin of proximal bicipital crest of humerus |
| *M. subscapularis* | Lateral and medial surfaces of cranial scapula | Ventral tubercle of humerus |
| *M. subcoracoideus* | Dorsolateral aspect of internal rostral spine of sternum, medial aspect of sternocoracoclavicular membrane | Ventral tubercle of humerus |
| *M. deltoideus pars major* | Medial aspect of acrocoracoid process of coracoid | Dorsocaudal margin of proximal humeral shaft |
| *M. deltoideus pars minor* | Lateral aspect of acrocoracoid process of coracoid, acromial process of furcula | Proximodorsal margin of humeral head |
| *M. deltoideus pars propatagialis* | Dorsomedial aspect of acromial process of furcula | Becomes propatagial ligament |
| *Propatagial ligaments* | M. deltoideus pars propatagialis | Radiale (propatagial ligament), distal portion of belly of M. extensor carpi radialis (elbow-limiting ligament) |
| *M. coracobrachialis cranialis* | Acrocoracoid process of coracoid | Dorsocranial humerus |
| *M. coracobrachialis caudalis* | Ventral aspect of craniolateral process of sternum, ventrolateral aspect of lateral process of coracoid, fascia of M. supracoracoideus | Dorsal aspect of ventral tubercle of humerus |
| *M. scapulotriceps* | Lateral surface of cranial scapula | Olecranon of ulna |
| *M. humerotriceps* | Humeral shaft | Olecranon of ulna (Suzuki et al., 2014) |
| *M. biceps brachii* | Craniolateral aspect of omal end of coracoid (Suzuki et al., 2014, Hudson et al., 1972), bicipital crest of humerus | Caudal aspect of proximal radius, cranial aspect of proximal ulna |
| *M. expansor secundariorum* | Distal portion of M. latissimus dorsi pars caudalis, caudolateral edge of M. subcoracoideus (Hudson et al., 1972), ventral edge of M. scapulohumeralis caudalis | Proximal three secondary remiges |
| *M. brachialis* | Brachial muscle fossa of distal humerus | Brachial muscle impression of proximal ulna |
| *M. pronator superficialis* | Ventral epicondyle of humerus (Suzuki et al., 2014) | Distal third of caudal radial shaft |
| *M. pronator profundus* | Ventral epicondyle of humerus (Suzuki et al., 2014) | Halfway down caudal radial shaft |
| *M. entepicondylo-ulnaris* | Fused with tendon of M. pronator profundus (Suzuki et al., 2014, Hudson et al., 1972) | Proximal third of ventral margin of ulnar shaft |
| *M. flexor digitorum superficialis* | Ventral epicondyle of humerus (Suzuki et al., 2014) | Ventral surface of distal phalanx of major digit (Suzuki et al., 2014), ventral margin of ulnare |
| *M. flexor digitorum profundus* | Ventral margin of proximal ulnar shaft | Alular phalanx, ventral surface of distal phalanx of major digit (Suzuki et al., 2014) |
| *M. flexor carpi ulnaris* | Ventral epicondyle of humerus (Suzuki et al., 2014) | Ventrocaudal ulnare, secondary remiges |
| *M. ulnometacarpalis ventralis* | Ventral aspect of distal ulna | Craniodorsal aspect of proximal carpometacarpus (Suzuki et al., 2014, Hudson et al., 1972) |
| *M. extensor carpi radialis* | Dorsal epicondyle of humerus | Cranioproximal aspect of alular metacarpus (Suzuki et al., 2014) |
| *M. supinator* | Dorsal epicondyle of humerus | Craniodorsal margin of radial shaft |
| *M. extensor digitorum communis* | Proximal ulna (Suzuki et al., 2014), dorsal epicondyle of humerus | Caudodorsal alular digit, base of first phalanx of major digit (Suzuki et al., 2014, Hudson et al., 1972) |
| *M. ectepicondylo-ulnaris* | Dorsal epicondyle of humerus (Suzuki et al., 2014) | Craniodorsal midshaft of ulna |
| *M. extensor carpi ulnaris* | Fascia of M. ectepicondylo-ulnaris | Caudal margin of major metacarpus (Suzuki et al., 2014) |
| *M. extensor longus alulae* | Dorsal aspect of proximal ulna, caudal margin of proximal radial shaft | Proximodorsal aspect of alular metacarpus (Suzuki et al., 2014) |
| *M. extensor longus digiti majoris* | Caudoventral margin of distal third of radius | Second phalanx of major digit (Suzuki et al., 2014) |
| *M. ulnometacarpalis dorsalis* | Distal ulnar shaft and dorsal aspect of ulnare | Caudal aspect of minor metacarpus |
| *M. abductor alulae* | Distal tendon of M. extensor carpi radialis (Hudson et al., 1972) | Cranial margin of ventral alular phalanx (Suzuki et al., 2014) |
| *M. flexor alulae* | Ventral aspect of alular process of carpometacarpus | Caudoventral margin of alular phalanx (Suzuki et al., 2014) |
| *M. adductor alulae* | Cranial margin of proximal major metacarpus | Alular quills (Suzuki et al., 2014), caudal margin of alular phalanx |
| *M. extensor brevis alulae* | Proximodorsal aspect of alular metacarpus | Cranial margin of alular phalanx |
| *M. abductor digiti majoris* | Ventral margin of major metacarpus | Cranial margin of proximal end of first phalanx of major digit (Suzuki et al., 2014) |
| *M. interosseus dorsalis* | Caudodorsal margin of major metacarpus, craniodorsal margin of minor metacarpus | Proximodorsal surface of minor digit phalanx (Suzuki et al., 2014), our observation. Anterodorsal edge of second phalanx of major digit to base of third phalanx (anterior branch), base of second phalanx of major digit (posterior branch) (Hudson et al., 1972) |
| *M. interosseus ventralis* | Caudoventral margin of major metacarpus, cranioventral margin of minor metacarpus | Proximal and distal phalanges of major digit (Suzuki et al., 2014) |
| *M. flexor digiti minoris* | Caudal margin of minor metacarpus | Caudal edge of minor digit phalanx |
